# Making use of longitudinal information in pattern recognition

**DOI:** 10.1002/hbm.23317

**Published:** 2016-07-25

**Authors:** Leon M. Aksman, David J. Lythgoe, Steven C.R. Williams, Martha Jokisch, Christoph Mönninghoff, Johannes Streffer, Karl‐Heinz Jöckel, Christian Weimar, Andre F. Marquand

**Affiliations:** ^1^ Department of Neuroimaging Institute of Psychiatry, Psychology & Neuroscience, King's College London London United Kingdom; ^2^ Department of Neurology University Hospital Essen, University of Duisburg‐Essen Essen Germany; ^3^ Department of Diagnostic and Interventional Radiology and Neuroradiology University Hospital Essen, University of Duisburg‐Essen Essen Germany; ^4^ Janssen‐Pharmaceutical Companies of Johnson & Johnson Janssen Research and Development Beerse Belgium; ^5^ Institute for Medical Informatics, Biometry and Epidemiology, University Hospital of Essen, University Duisburg‐Essen Germany; ^6^ Donders Institute for Brain, Cognition and Behaviour, Radboud University Nijmegen the Netherlands

**Keywords:** pattern recognition, longitudinal studies, principal component analysis, mild cognitive impairment, dementia, structural MRI, classification, support vector machines

## Abstract

Longitudinal designs are widely used in medical studies as a means of observing within‐subject changes over time in groups of subjects, thereby aiming to improve sensitivity for detecting disease effects. Paralleling an increased use of such studies in neuroimaging has been the adoption of pattern recognition algorithms for making individualized predictions of disease. However, at present few pattern recognition methods exist to make full use of neuroimaging data that have been collected longitudinally, with most methods relying instead on cross‐sectional style analysis. This article presents a principal component analysis‐based feature construction method that uses longitudinal high‐dimensional data to improve predictive performance of pattern recognition algorithms. The method can be applied to data from a wide range of longitudinal study designs and permits an arbitrary number of time‐points per subject. We apply the method to two longitudinal datasets, one containing subjects with mild cognitive impairment along with healthy controls, the other with early dementia subjects and healthy controls. Across both datasets, we show improvements in predictive accuracy relative to cross‐sectional classifiers for discriminating disease subjects from healthy controls on the basis of whole‐brain structural magnetic resonance image‐based voxels. In addition, we can transfer longitudinal information from one set of subjects to make disease predictions in another set of subjects. The proposed method is simple and, as a feature construction method, flexible with respect to the choice of classifier and image registration algorithm. *Hum Brain Mapp 37:4385–4404, 2016*. © **2016 Wiley Periodicals, Inc.**

## INTRODUCTION

Longitudinal studies aim to follow a set of subjects, making repeated observations of the same variables over time. They have been widely used in medical studies to provide more sensitive detection of disease effects by focussing on within‐subject changes in clinical groups [Fitzmaurice and Ravichandran, 2008]. Such study designs are increasingly being used in neuroimaging research, often with the goal of gathering imaging biomarkers for assessing disease progression and, potentially, response to therapy [Bernal‐Rusiel et al., 2013; Chen et al., 2012; Douaud et al., 2013; Guillaume et al., 2014]. At the same time, pattern recognition algorithms have been adopted by the neuroimaging community to make use of such biomarkers to make individualized predictions of subjects' current or future disease state [Klöppel et al., 2012; Orrù et al 2012; Wolfers et al., 2015]. At present, however, most pattern recognition‐based analyses use cross‐sectional data or analyze longitudinal data in a cross‐sectional manner and therefore do not capitalise on the full value of longitudinal designs. This article describes a novel method of constructing features from high‐dimensional longitudinal data such as imaging biomarkers that solves this problem. Our method is applicable to most forms of high‐dimensional data; here, we have used structural magnetic resonance images (MRI), with the goal of discriminating subjects with mild cognitive impairment (MCI) from healthy controls (HCs).

Structural MRI is a popular source of imaging biomarkers due to the high spatial resolution images that can be obtained in a safe and clinically acceptable manner. Traditionally, cross‐sectional classifiers that only use data from a single time‐point have performed reasonably well with such data on certain problems. For example, in the case of discriminating subjects with Alzheimer's Disease (AD) from HCs using structural MR images, the pathology of the disease is severe enough that there are usually significant volumetric differences between age‐matched samples from these two groups to discriminate subjects cross‐sectionally. In a direct and controlled comparison, it was shown that both radiologists and automated methods perform comparably, achieving roughly 90% accuracy in both cases for discriminating subjects with sporadic AD from controls [Klöppel et al., 2008].

Given promising results discriminating such severe forms of neurodegeneration from controls using structural MRI, researchers have attempted to discriminate earlier forms of neurodegeneration as well as subjects who are more likely to progress to dementia in the future [Frisoni et al., 2010]. MCI, in particular, has been a focus of intense study in recent years as it is widely believed to be the best stage for interventional treatments aimed at delaying or preventing progression to AD [Morris et al., 2001]. Discriminating MCI from controls, particularly with structural MRI, has proven to be more difficult however [Chu et al., 2012; Mönninghoff et al., 2015]. In addition to the problem of head size and shape differences between subjects that pertains to much of neuroimage‐based analysis, the study of early stage diseases and disease precursors is often confounded by demographic factors such as age, gender, and education level [Bakkour et al., 2013; Dukart et al., 2013]. Due to such sources of inter‐subject variance, the more subtle nature of the pathology of MCI may be hard to discern from healthy aging via volumetric biomarkers derived from structural MRI. This in turn may explain the poorer performance in discriminating MCI from controls using pattern recognition‐based classifiers, commonly reported thus far [Orrù et al., 2012].

In cases of high inter‐subject variation, longitudinal study designs provide a means of increasing the sensitivity of detecting disease effects of interest by tracking intra‐subject differences over time. Such study designs are particularly appropriate for studying neurodegenerative diseases as they are, by definition, a class of diseases marked by progressive loss of structure and/or function [Franke and Gaser, 2012; Misra et al., 2009; Risacher et al., 2009]. Raz and Lindenberger [2011] strongly advocate the increased use of intra‐subject longitudinal information rather than inter‐subject cross‐sectional information to study aging. Several recent neuroimaging studies have done so in a mass univariate context. Zipunnikov et al. [2014] developed an efficient method for handling very high dimensional neuroimaging data using dimensionality reduction, building a mixed effects model that decomposes the variability of repeated observations into subject specific cross‐sectional, longitudinal and exchangeable visit‐to‐visit variabilities. Ziegler et al. [2015], applied Bayesian linear mixed effects modelling to structural neuroimaging data from dementia subjects as well as older healthy subjects, comparing temporal trajectory models with varying model orders for fixed and random effects in a principled manner, through the use of model evidence. Such mass univariate methods hold great value for explaining the sources of variance and localizing disease effects in a cohort of study subjects. However, there is no mechanism for understanding how well such models generalize to unseen data. Pattern recognition‐based approaches, in contrast, aim to assess predictive performance on held out samples, making them better suited for clinical applications.

Other neuroimaging methods have made use of longitudinal information at the image registration stage, thereby making them applicable to pattern recognition as feature construction methods [Holland and Dale, 2011; Leung et al., 2012]. Ashburner and Ridgway [2013] developed a group‐wise longitudinal registration technique which creates an intra‐subject template using two or more images of a subject. In the case of two images per subject, longitudinal features for pattern recognition can then be computed that quantify the contraction or expansion of each voxel from baseline to follow‐up time‐point. In one of the few direct applications of pattern recognition to longitudinal neuroimaging data, Gray et al. [2012] used a longitudinal registration‐based approach to classify dementia. The authors non‐rigidly registered the 12‐month follow‐up FDG‐PET images of subjects to their corresponding baseline images. The authors show that longitudinal features, encoding the change from baseline to follow‐up, perform worse in two class classifications involving AD, MCI, and HCs than cross‐sectional features. However, concatenating longitudinal features with cross‐sectional features resulted in a small improvement in balanced accuracies compared to using purely cross‐sectional features.

Longitudinal information has also been integrated into a classifier at the level of the algorithm itself, by providing subjects' cross‐sectional images from multiple time‐points as inputs to the algorithm. Chen and DuBois Bowman [2011] presented a method that generalizes the optimization problem of the popular support vector machine (SVM) by constructing a support vector classifier (SVC) that includes a linear combination of an arbitrary number of cross‐sectional time‐points from all subjects. At each iteration, their algorithm proceeds by optimizing the support vector (SV) coefficients under fixed time‐point weightings followed by optimizing the time‐point weightings, common across all subjects, under the updated SV coefficients, until convergence.

In this work, we propose a method for incorporating longitudinal information into a classifier via feature construction rather than modifying a particular pattern recognition algorithm's optimization problem or relying on specialized registration techniques. We project cross‐sectional data onto a low‐dimensional linear subspace formed by performing principal component analysis (PCA) on a matrix of coefficients describing longitudinal changes. Such low‐dimensional subspaces, formed using training data, have been broadly used in the context of facial recognition problems [Belhumeur et al., 1997; Chen et al., 2005; He et al., 2005; Turk and Pentland, 1991; Zhao et al., 2007]. In the context of neuroimaging, Wolz et al. [2010] learned a low‐dimensional manifold using both longitudinal and cross‐sectional information to classify dementia, using two‐sample difference images to represent longitudinal change. Our method is similar to this approach but is applicable to both “balanced” (fixed number of samples per subject and fixed time interval between samples) and “unbalanced” designs (varying number of samples per subject or varying intervals between samples) and is able to make use of an arbitrary number of time‐points per subject. Importantly, it also has the ability to transfer longitudinal information from one set of subjects to make individualized predictions for subjects from another set. Furthermore, the method is fast and relatively simple, having only one parameter that is tuned via cross‐validation. As a feature construction method it is not restricted to any neuroimaging modality, image registration technique or pattern recognition algorithm.

Here, we demonstrate the method using a linear SVC and structural MRI‐based biomarkers on the tasks of discriminating subjects with MCI from HCs and early dementia from HCs using two independent datasets. We compare the accuracies obtained using longitudinal features to those obtained using only cross‐sectional features and predict that longitudinal information will yield performance improvements that depend on the type of longitudinal study design used and number of samples per subject that are available.

## MATERIALS AND METHODS

### Subjects

For the first dataset, we used clinical and imaging data derived from a substudy of the Heinz Nixdorf Recall (HNR; Risk Factors, Evaluation of Coronary Calcium and Lifestyle) study. The HNR study is a population‐based prospective cohort study with subjects randomly selected from mandatory city registries in Germany. Study methods have been previously described in detail [Schmermund et al., 2002; Stang et al., 2005]. Briefly, 4,814 participants 45 to 75 years of age were enrolled between 2000 and 2003 in the Ruhr area in Germany. After the baseline examination participants were followed over a five year period when a second examination was conducted. The second examination (response rate: 90.2%) included a short cognitive performance assessment (for details regarding participants and drop‐outs see Dlugaj et al. [2010]), which was accomplished in 4,086 study participants. At this follow‐up time‐point, a random sample of participants (aged 50–80) with impaired short cognitive performance assessment results (*n* = 701) and age appropriate short cognitive performance assessment results (*n* = 316) were invited to a detailed neuropsychological and neurological examination to assess MCI and its subtypes for inclusion in the HNR substudy (HNRS) [Dlugaj et al., 2010]. The neuropsychological examinations performed and MCI diagnosis criteria used in this study are detailed in the Supporting Information.

In total, the HNRS consisted of 148 MCI cases identified and matched, at the five year follow‐up of the total HNR cohort, to 148 HCs according to age, sex, and education. Participants were examined with MRI at the substudy baseline (starting at the five year follow‐up of the total cohort) and at the substudy 2.5 years follow‐up. A second follow‐up with MRI, approximately 5.5 years after substudy baseline, is being conducted, with data not yet available. All participants gave their written informed consent. The study was approved by the local institutional ethical committee and followed established guidelines of good epidemiological practice.

The second dataset used was the OASIS longitudinal sample, the full details of which have been reported previously [Marcus et al., 2010]. The longitudinal MRI data consisted of 150 subjects aged 60 to 96, recruited primarily through media appeals and word of mouth. Each of the 150 study subjects had at least two separate visits in which clinical data and MRI data were collected. Clinical data consisted of dementia status, as measured by the Clinical Dementia Rating (CDR) scale. The CDR scale ranges from 0 for no cognitive impairment, to 0.5 for very mild dementia, one for mild dementia, two for moderate dementia, and three for severe dementia. Subjects with a primary cause of dementia other than AD (e.g., vascular dementia, primary progressive aphasia), active neurological or psychiatric illness (e.g., major depression), serious head injury, history of clinically meaningful stroke, and use of psychoactive drugs were excluded, as were subjects with gross anatomical abnormalities evident in their MRI images (e.g., large lesions, tumors).

### Data Acquisition and Quality Control

For the HNRS dataset, MR examinations of the head were performed in all study participants with a confirmed diagnosis of MCI and in their matched controls. Each MR examination was performed on a single 1.5T MR scanner (Magnetom Avanto, Siemens Healthcare, Erlangen). The MR scanner was equipped with a 12‐channel receive‐only matrix head coil provided by the vendor. A sagittal 3D Fast Low Angle SHot T1 weighted image (TR = 40 ms; TE = 5 ms; flip angle = 40°; matrix size = 256 × 256 with 176 1.0 mm thick slices; FoV = 26 mm × 26 mm; bandwidth = 160 Hz/pixel) was acquired and used in this analysis. In addition, fluid‐attenuated inversion recovery and T2 weighted sequences were also acquired for standard radiological examination.

For the OASIS dataset, all of the sagittal 3D magnetization prepared rapid acquisition of gradient echo images (TR = 9.7 ms; TE = 4 ms; TI = 20 ms; TD = 200 ms; flip angle = 10°; matrix size = 256 × 256 with 128 1.0 mm thick slices; FoV = 25.6 mm × 25.6 mm) were also acquired on a Siemens MR system operating at 1.5T (Magnetom Vision, Siemens Healthcare, Erlangen). At each visit, three or four T1‐weighted images were acquired for each subject.

For both studies, all T1 weighted images were visually inspected. Images with artefacts or any pathology not associated with dementia, such as signs of stroke, were discarded from further analysis.

### Data Pre‐Processing

The same image segmentation and image registration procedure described here was performed for both datasets, which were analyzed separately. Subjects' baseline and follow‐up images meeting the quality control standards described above were segmented into gray matter and white matter using SPM8's “New Segment” procedure. Gray matter and white matter tissue class images were registered to a common inter‐subject space, by creating a study specific template using SPM8's DARTEL registration procedure [Ashburner, 2007]. We formed the template using images from all time‐points. In this study, we used Jacobian determinant images from the warping of each subject's gray matter and white matter images to the common template. While our method is generic and can be applied to any type of feature (e.g., modulated gray‐matter images), Jacobian determinant images have shown promise in discriminating neurodegeneration by allowing a comparison of the expansion and contraction of voxels across and within subjects [Anderson et al., 2012; Hua et al., 2009, 2010; Studholme et al., 2004].

A whole brain mask was applied to exclude the extracerebral voxels. To form the mask, we segmented the MNI152 brain with the same “New Segment” procedure, then registered the resulting gray matter image to the study template's gray matter tissue class image using FMRIB's Nonlinear Image Registration Tool [Andersson et al., 2007a, 2007b]. We applied the resulting warp to the Harvard‐Oxford subcortical atlas [Desikan et al., 2006] that has been affine‐registered to MNI152, available in FSL [Smith et al., 2004]. We then formed the mask as a binary image consisting of all atlas regions excluding the brainstem and cerebellum. Masked images, retaining 356,365 voxels in all cases, were then reshaped to form high‐dimensional data samples used in subsequent analysis.

For comparison to our proposed features, we also created features quantifying longitudinal change using a within‐subject diffeomorphic registration method developed by Ashburner and Ridgway [2013] and available in SPM12b. We coregistered each subject's baseline image to its follow‐up, generating a temporal midpoint image in the process as well as Jacobian determinant images. We then performed the same inter‐subject procedure on the midpoint images as before, segmenting them using SPM8's “New Segment” procedure and using DARTEL to form an inter‐subject template with the gray and white matter tissue class images from the segmentation. Rather than using the Jacobian determinant images from the warping of each subject's midpoint to the common template, we warped the Jacobian determinant images from the intra‐subject registration to the common template. A whole brain mask was also applied to these images in the same manner described above. We used these registered intra‐subject Jacobian determinant images as longitudinal features, thereby quantifying the amount of expansion or contraction that each voxel undergoes due to within‐subject coregistration.

### Method Overview

We introduce a model for subject specific changes in samples as polynomial functions of time in the general case of an unbalanced longitudinal design. Using the coefficient matrices from the solutions of these subject specific models, we create a transformation into a subspace describing intra‐subject longitudinal change that is common across subjects. We form features for classification by projecting cross‐sectional samples onto this longitudinal subspace. For the special case of two samples per subject, for both balanced and unbalanced designs, we show that the intra‐subject difference between samples, scaled by the time differences between samples, is equivalent to the matrix of coefficients of linear change over time (i.e., the slope coefficients), leading to a simple method for creating linear transformations that model longitudinal changes.

We will make use of several sets in this section. The longitudinal set $\bar{L}$ of subjects will be defined as the set of subjects for whom the necessary number of longitudinal time‐points is available. As we use cross‐validation to evaluate a classifier's performance on unseen data, we define the classification set C as the set that is split, at each cross‐validation fold, into a training set N of subjects used to train a classifier and a set T of subjects used to test the classifier's ability to generalize to unseen data. Additionally we define the longitudinal training set L as the set difference between $\bar{L}$ and T, $L=\bar{L}\backslash T=\left\{x\;\in \bar{L}\;\right|\;x\;\notin \;T\}$, that is, the longitudinal set subjects that are not being held out for testing in a given cross‐validation fold.

### Longitudinal Trajectory Model

We assume that a regression model can be estimated independently for each of the l subjects in the longitudinal training set L, such that for subject i, having $m_{i}$ samples of dimensionality D, we have $X_{i}=Z_{i}B_{i}$, where $X_{i}$ is an $m_{i}\times D$ matrix of the subject's samples, ${\;Z}_{i}$ is an $m_{i}\times (P+1)$ longitudinal design matrix with chosen model order P (with a constant term included as the extra dimension) and $B_{i}$ is an (P+1)×D matrix of voxel trajectory coefficients for that subject. Using this assumed model form, Appendices A and B derive expressions for $B^{\left(1\right)}$, the matrix of first‐order (slope) coefficients of voxels' longitudinal trajectories across subjects and image dimensions, for the general case of an unbalanced design (varying numbers of samples per subject or varying time intervals, Appendix A) and the special case of two time‐point per subject balanced and unbalanced longitudinal designs (with fixed or varying time intervals, respectively, Appendix B). We consider this special case separately as longitudinal data in neuroimaging are often limited to the minimum of a single baseline and follow‐up image per subject. Due its simplicity and importance in our analysis, Eq. (B.4), the expression for $B^{\left(1\right)}$, the matrix of linear (slope) coefficients across subjects and voxels in the balanced two sample case, is restated here as

(1) $$D_{L}≜\frac{1}{t_{\Delta }}{(X}_{L}\left(t_{2}\right)-X_{L}\left(t_{1}\right))=B^{\left(1\right)}$$

where we have defined the matrix $D_{L}$ as the intra‐subject (longitudinal) differences between the baseline time‐point's samples $X_{L}\left(t_{1}\right)$ and the follow‐up time‐point's samples $X_{L}\left(t_{2}\right)$, scaled by $t_{\Delta }$, the fixed time interval between samples.

In this article, we use the $B^{\left(1\right)}$ matrix (derived from either the general or special cases described above) to create projections that capture a subspace of linear longitudinal change that is common across subjects. We note that one can model non‐linear longitudinal changes by building a subspace using higher order coefficient matrices ( $B^{\left(2\right)},\;\ldots ,\;B^{\left(P\right)},\;P>1)$ based on the method described in Appendix A, although we focus on linear subspaces by fitting first‐order models ( P=1) here. However, our approach easily accommodates datasets with a greater number of follow‐up timepoints.

### Longitudinal Subspace Formation

To generalize to unseen data, we use PCA to create an orthogonal projection of the l D‐dimensional samples in the l×D matrix $B^{\left(1\right)}$ onto a low‐dimensional linear subspace that describes most of the variance in the data. The projection matrix, $U_{k}$, is composed of a small set of k principal components (PCs) of $B^{\left(1\right)}$, that we term “eigenslopes” in the spirit of Turk and Pentland [1991], such that $U_{k}$ is of size D×k, with k≪D. We have chosen to use PCA as it is a computationally efficient and linear technique, the latter property allowing for easier interpretation of results. Section 12.1.4 of Bishop [2007] describes the PCA procedure we used to compute $U_{k}$, with the number of retained PCs k chosen as described in the Nested Cross‐Validation section.

We refer to the PCA performed on the coefficient matrix $B^{\left(1\right)}$ described by Eq. (A.6) for the general case of an unbalanced design as Longitudinal Trajectory Coefficient PCA (LTC‐PCA). We refer to the PCA performed on the matrix $D_{L}$, shown in Appendix B to be an approximation for $B^{\left(1\right)}$ in the special case of two time‐points per subject (for both balanced and unbalanced designs), as Longitudinally Matched PCA (LM‐PCA).

### Within‐Set Prediction Problem

**Figure 1:**
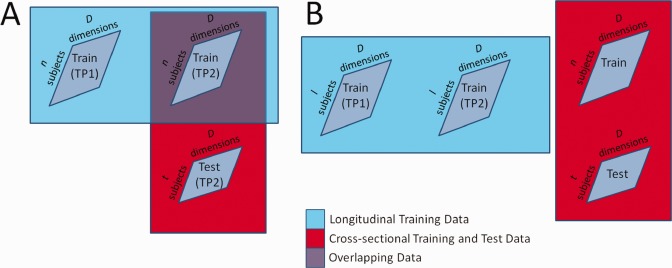
**Illustration of Within‐Set Prediction and Information Transferring Prediction problems**. Illustration of longitudinal and cross‐sectional data used in the Within‐Set Prediction (Panel **A**) and Information Transferring Prediction (Panel **B**) problems when there is data from two time‐points (TP1 and TP2). Panel A depicts the Within‐Set Prediction problem of predicting disease state at a follow‐up time‐point (TP2) using longitudinal information from both time‐points. In this case, the longitudinal training subject set (data in blue) matches the classification training subject set (data in the top part of the red area), with overlapping data shown in purple. To form the proposed features as in Eq. (2), the cross‐sectional training data (in purple) and test data (non‐overlapping area in red) is projected onto a subspace formed using the longitudinal training data (blue area). Graphic B depicts the Information Transferring Prediction problem, where the longitudinal subject set and classification subject sets are disjoint. In this case, there is no overlap in data between the longitudinal data (blue area) used to form the subspace in Eq. (2) and the classification data (both training and test data, red area) being projected. Here, the cross‐sectional data may come from a baseline or follow‐up time‐point.

When the longitudinal set $\bar{L}$ and the classification set C are equal all subjects for whom we are interested in making predictions via cross‐validation have the necessary longitudinal samples. At each cross‐validation fold, we form a training set N and test set T as subsets of C and form the projection $U_{k}$ using the longitudinal information of the subjects in N, which is equivalent to the longitudinal training set L in this case. This is a straightforward application of our method, which seeks to answer whether longitudinal information from a set of subjects can improve predictions for subjects within the set (Fig. 1A).

### Information Transferring Prediction Problem

In addition to the Within‐Set Prediction problem, our method can be used to transfer longitudinal information from one set of subjects to another. In this case, the longitudinal set $\bar{L}$ and the classification set C are disjoint, meaning they have no subjects in common. It follows that the longitudinal training set L, used to form the longitudinal transform $U_{k}$, is disjoint from both the training set N and test set T at each cross‐validation fold. This problem illustrates that our approach can be used to form a longitudinal subspace with one set of subjects to make diagnostic predictions for another subject set (Fig. 1B).

### Feature Formation

For both cases described above, we form training and test feature matrices, used in classification, by projecting the cross‐sectional samples of the training and test subjects' samples at a particular time‐point t onto a rank k longitudinal space described by $U_{k}{U_{k}}^{T}$. Note that the difference between the two cases described above is whether subject set L, whose longitudinal data is used to form $U_{k}$, is equal to or disjoint from set N.

For either case, at each cross‐validation fold the features used in classifier training and testing have the form

$$X_{train\;}=X_{N}(t)U_{k}U_{k}^{T}$$

(2) $$X_{test}=X_{T}(t)U_{k}U_{k}^{T}$$

Here, we have introduced $X_{N}(t)$ and $X_{T}(t)$ as, respectively, matrices of cross‐sectional data for the training and test sets at a particular time‐point t, such as at baseline or follow‐up. We use $X_{train}$ as an input feature matrix to train a classifier to predict $y_{train}\;\in \;\{1,-1\}$, the binary class labels of the training set at a given time‐point. We test the trained classifier's predictive performance using $X_{test}$ as an input feature matrix to predict $y_{test}\;\in \;\{1,-1\}$, the binary class labels of the test set at a given time‐point. In this article we set $X_{N}(t)=X_{N}(t_{2})$ and $X_{T}(t)=X_{T}(t_{2})$ when we predict the follow‐up class labels using projected follow‐up cross‐sectional samples and $X_{N}(t)=X_{N}(t_{1})$ and $X_{T}(t)=X_{T}(t_{1})$ when we predict the baseline labels using projected baseline samples. It is also possible to make prognostic predictions of the follow‐up labels by projecting the baseline cross‐sectional data matrices $X_{N}(t_{1})$ and $X_{T}(t_{1})$ onto the longitudinal subspace. We have chosen to transform the features back into the original D×1 space by post‐multiplying by ${U_{k}}^{T}$, allowing an easier interpretation of the resulting model's D×1 weight vector as voxels in a masked brain image. Alternatively, to obtain low‐dimensional feature vectors, one may simply project the baseline or follow‐up features onto $U_{k}$ instead of $U_{k}U_{k}^{T}$.

**Figure 2:**
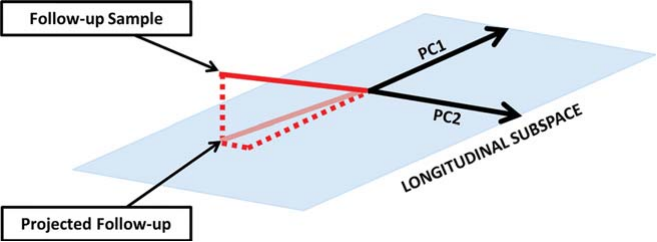
**Example of a 2D longitudinal subspace projection**. To gain intuition for the longitudinal projection described by Eq. (2), we consider a simple case of a sample vector (Follow‐up Sample) that lies in three dimensional space (an image consisting of three voxels) being projected onto the two dimensional plane described by two principal component vectors (PC1, PC2) extracted from a hypothetical coefficient matrix describing subjects' longitudinal changes. The projected sample (Projected Follow‐up) is thereby composed of the components of the sample vector that lie in the space of common longitudinal changes, described by the principal components.

**Figure 3:**
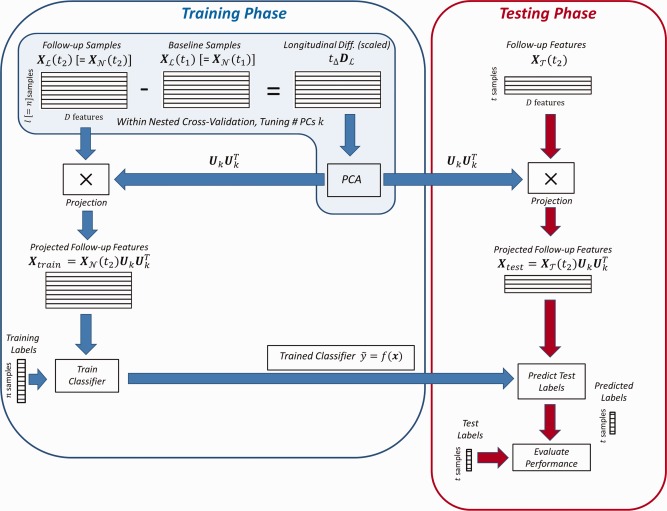
**Schematic of a cross‐validation fold forming LM‐PCA projected features, Within‐Set Prediction problem**. Schematic of a cross‐validation fold implementing LM‐PCA projected features, for the Balanced Within‐Set Prediction problem of classifying follow‐up time‐point disease label, as in Tables 1 and 3. We have emphasized that the longitudinal training set is equivalent to the classification training set in this case: the same follow‐up data used to form the longitudinal difference matrix [scaled by the time interval between scans as in Eq. (1)] is projected onto the longitudinal subspace in the training step.

**Figure 4:**
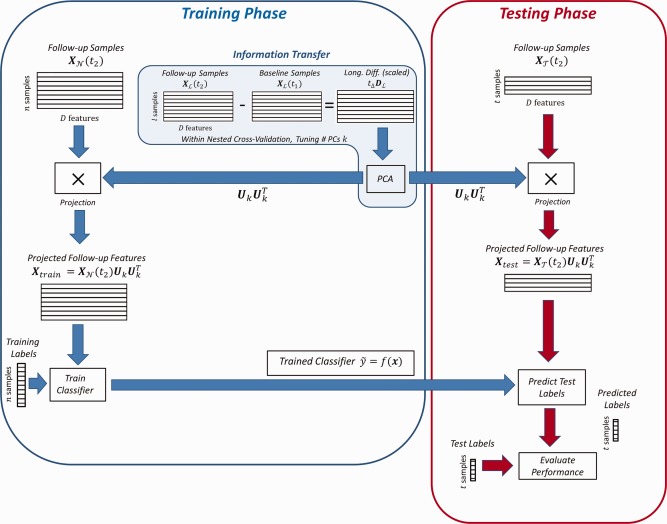
**Schematic of a cross‐validation fold forming LM‐PCA projected features, Information Transferring Prediction problem**. Schematic of a cross‐validation fold implementing LM‐PCA projected features, for the Balanced Information Transferring Prediction problem of classifying follow‐up time‐point disease label, as in Table 2. We have emphasized that the longitudinal and classification subject sets are disjoint in this case, with the data in the “Information Transfer” area in blue being used to form the longitudinal difference matrix [scaled by the time interval between scans as in Eq. (1)]. As a result, the follow‐up data that is projected onto the the longitudinal subspace differs from the follow‐up data used to form the longitudinal difference matrix.

Figure 2 helps provide an understanding of the projection described by Eq. (2) by means of a simple two‐dimensional longitudinal subspace example. The projected features can be seen as the components of cross‐sectional data that lie in a space of common longitudinal change described by the retained PCs (eigenslopes) in $U_{k}$. In the figure there are two retained PCs, forming a two‐dimensional plane on which a higher dimensional cross‐sectional (follow‐up) sample is projected. The schematics in Figures 3 and 4 depict the formation of training and test features at each cross‐validation fold for the Within‐Set Prediction and Information Transferring Prediction problems respectively, with matrix $U_{k}$ formed by performing LM‐PCA on balanced longitudinal design data in both cases.

### Kernel Formation

To keep the number of tuneable parameters to a minimum, we use a linear kernel of the form $K=XX^{\prime T}$, where X and $X^{\prime }$ may be a training or testing feature matrix. Without loss of generality, the kernel used during classifier training can be expressed as

(3) $$K=\left(X_{N}{(t)U}_{k}U_{k}^{T}\right){\left(X_{N}{(t)U}_{k}U_{k}^{T}\right)}^{T}=\left(X_{N}{(t)U}_{k}\right){\left(X_{N}{(t)U}_{k}\right)}^{T}$$

as $U_{k}$ is composed of a set of orthonormal vectors. Therefore, the effective dimensionality of the feature vectors is reduced from D to k by the PCA procedure described. Prior to use in training and testing, we mean‐centre the features using the training data.

### Classification Algorithm

As a feature construction method, our method can be used with a range of different classification algorithms. In this work we show results using the linear SVM algorithm, which has been widely applied to neuroimaging [Davatzikos et al., 2008; Mourão‐Miranda et al., 2005]. Lemm et al. [2011] and Orrù et al. [2012] provide a thorough explanation and review of the algorithm's application to disease state classification using neuroimaging. We used the LIBSVM [Chang and Lin, 2011] implementation of the C cost SVC described above [Cortes and Vapnik, 1995]. We used a fixed value of C=1 throughout all classifications. Figure S1 in the Supporting Information shows that this choice provides near optimal prediction performance for the datasets we consider here because it lies in a stable region of maximal classifier performance across the three types of features used in Table 1. However, in some cases it may be necessary to optimize C by cross‐validation to obtain optimal performance. This may be the case if the input vectors are already relatively low‐dimensional (e.g., region of interest [ROI] summary measures).

**Table 1:** HNRS Dataset, Balanced Within‐Set Prediction, discriminating MCI subjects versus HC subjects with two longitudinal time‐points per subject for all subjects (classification subject set equal to longitudinal subject set in this case)

Features	Bal. accuracy (%)	Sensitivity (%)	Specificity (%)	PPV (%)	NPV (%)	ROC AUC
Predicting Follow‐up Class Label (MCI *n* = 24, HC *n* = 23) using same subjects in longitudinal subject set						
Follow‐up	53.2	54.2	52.2	54.2	52.2	0.639
Longitudinal coregistration^a^	59.6	58.3	60.9	60.9	58.3	0.574
Follow‐up, Baseline Subspace Projected	59.7	54.2	65.2	61.9	57.7	0.578
Follow‐up, Follow‐up Subspace Projected	59.6	58.3	60.9	60.9	58.3	0.627
Follow‐up, LM‐PCA Projected	74.3	83.3	65.2	71.4	78.9	0.774

a Features based on Ashburner and Ridgway [2013].

Statistically significant balanced accuracy, permutation test *P*‐value < 0.05.

Statistically significant balanced accuracy, permutation test *P*‐value < 0.01.

### Nested Cross‐Validation

The classification model as described has only one parameter that must be tuned via nested cross‐validation: the number of PCs k that are retained to form the low‐rank projection matrix $U_{k}{U_{k}}^{T}$. We used an outer leave‐one‐out cross‐validation (LOO‐CV) scheme to make predictions via the optimized parameter k and an inner LOO‐CV scheme to optimize k. Within each inner CV fold, the model was trained and tested across a range of k values corresponding to specified amounts of variance explained by the retained PCs. For a desired fraction of explained variance $p_{var},\;k$ is chosen as the minimum such that $\sum_{i=1}^{k}\lambda_{i}/\sum_{i=1}^{n}\lambda_{i}\;\geq \;p_{var}$, where $\lambda_{i}$ is the *i*th highest eigenvalue of the covariance matrix used in the PCA [Bishop, 2007]. In this study, we varied explained variance from 5% to 95% in increments of 5%. The final optimized value of k, used in the outer CV fold, was based on the explained variance value that had the highest balanced accuracy across all inner CV folds.

The balanced accuracy, introduced by Brodersen et al. [2010], is the average of Sensitivity=TP/(TP+FN) and Specificity=TN/(TN+FP). Here TP, FP,  TN,  FN are the number of true positives (correctly classified disease subjects), false positives (incorrectly classified control subjects), true negatives (correctly classified control subjects) and false negatives (incorrectly classified disease subjects), respectively. This metric, (Sensitivity+Specificity)/2, was used rather than the overall accuracy, defined as (TP+ TN)/(TP+TN+FP+FN), as it is less sensitive to imbalanced class sizes.

### Method Comparison and Performance Evaluation

In addition to the balanced accuracy, sensitivity, and specificity measures of classifier performance already mentioned, we provide measures of positive predictive value ( PPV), negative predictive value ( NPV) and a summary of the receiver operating characteristic's area under the curve ( ROC AUC). PPV is the proportion of disease state (+1 class) predictions which are correct, PPV=TP/(TP+FP). NPV is the proportion of healthy state (−1 class) predictions which are correct, NPV=TN/(TN+FN). The ROC AUC plots the true positive versus false positive rate as the classifier's decision threshold is varied and is considered to be a robust measure of classifier performance in the sense that it is independent of an arbitrary choice of decision threshold.

To compare the performance of a classifier that uses our proposed features, we also build a cross‐sectional classifier using features based strictly on either the baseline or follow‐up imaging features, as appropriate, which reflects current practice. We also demonstrate the performance of longitudinal features formed using the within‐subject registration of Ashburner and Ridgway [2013], as described in the Data Pre‐processing section.

### Permutation Testing

Permutation tests were performed to assess the statistical significance of the balanced accuracies of each classifier, relative to chance. Each permutation test involved randomly rearranging the order of the elements in the vector of n subjects' class labels ${\left[y_{1},\ldots ,y_{n}\right]}^{T}$ to form a random vector of class labels that retains the original number of subjects in each class. Predicting these random class labels allowed us to build up null distributions of balanced accuracies for our proposed features and the comparison features. A *P*‐value was then estimated by dividing the number of balanced accuracies in the null distribution that match or exceed the true balanced accuracy by the total number of permutations. In this work we present results using null distributions built with 1,000 permutations of class labels [Jockel, 1986; Mourão‐Miranda et al., 2005; Nichols and Holmes, 2002].

### Model Interpretation: Weight Maps and Forward Maps

As the resulting SVM weight vector w is in the original D‐dimensional feature space, we can visualize the weights of the classifier. In general, the weight vector is calculated as

(4) $$w=\sum_{i=1}^{n}\alpha_{i}y_{i}\phi (x_{i})=\sum_{i=1}^{n}{\bar{\alpha }}_{i}\phi (x_{i})$$

where $\phi (x_{i})$ is the feature vector in the space implicitly defined by the chosen kernel and the ${\bar{\alpha }}_{i}$'s are the signed versions of the $\alpha_{i}\geq 0$ SV weights from the SVM optimization [Chang and Lin, 2011]. As we use linear features in this work, we have $\phi \left(x_{i}\right)=x_{i}$. Equation (4) can then be expressed as

(5) $$w={\hat{X}}^{T}\bar{\alpha }$$

where $\hat{X}$ is the mean‐centred version of a training feature matrix and $\bar{\alpha }$ is the n×1 vector of ${\bar{\alpha }}_{i}$'s, with entries corresponding to the rows of $\hat{X}$.

In addition to visualizing the classifier weights, we can also build a “forward map,” defined in Haufe et al. [2014], in the following way:

(6) $$a\propto Cov\left(X,\;\tilde{y}\right).$$

Here, a is the D×1 mapping that encodes the n×1 classifier function output $\tilde{y}$ in the feature space.

The weight map defined by Eq. (5) is useful for understanding the contribution of each feature toward the prediction of a sample's diagnostic label. As discussed in Haufe et al. [2014], interpretation of such maps should be done with care as a feature may have a high weight by virtue of a group difference or as a result of high collinearity between features, for example, features may obtain a high weight to cancel out noise in other features. In contrast, the encoding weights from the covariance‐based forward map in Eq. (6) represent the group differences between classes, which are often of interest when interpreting a trained classifier. If the data are standardized, the forward maps are equivalent to “structure coefficients” widely used in multiple linear regression [Kraha et al., 2012]. When discriminating a positively labelled (disease) class from a negatively labelled (control) class, stronger positive values in a forward map indicate a stronger association of a region with the positive class while negative values indicate a stronger association with the negative class (alternatively, a weaker association with the positive class).

## RESULTS

The results in this section make reference to the two types of classification problems described in the Methods section, namely the Within‐Set Prediction and Information Transferring Prediction problems as well as the two types of PCA‐based projections: LTC‐PCA and LM‐PCA. We will refer to Balanced problems when the longitudinal subject set has both fixed follow‐up times and a fixed number of scans per subject (in this case two) and Unbalanced problems when the longitudinal subject set has either varying follow‐up times or a varying number of scans per subject. We applied our proposed projection method (LM‐PCA and/or LTC‐PCA) as well as several comparison methods to five classification problems across two datasets (described below, with results shown in Tables 1, 2, 3, 4).

**Table 2:** HNRS Dataset, Balanced Information Transferring Prediction, discriminating MCI subjects versus HC subjects with two longitudinal time‐points per subject in longitudinal subject set (which is the same set of subjects used in Table I)

Features	Bal. accuracy (%)	Sensitivity (%)	Specificity (%)	PPV (%)	NPV (%)	ROC AUC
Predicting Baseline Class Label (MCI *n* = 79, HC *n* = 87) using disjoint longitudinal subject set (MCI *n* = 24, HC *n* = 23) from Table 1						
Baseline	49.1	43.0	55.2	46.6	51.6	0.474
Longitudinal coregistration^a^	—	—	—	—	—	—
Baseline, Baseline Subspace Projected	38.1	27.8	48.3	32.8	42.4	0.401
Baseline, LM‐PCA Projected	48.7	34.2	63.2	45.8	51.4	0.509
Predicting Follow‐up Class Label (MCI *n* = 10, HC *n* = 20) using disjoint longitudinal subject set (MCI *n* = 24, HC *n* = 23) from Table 1						
Follow‐up	45.0	20.0	70.0	25.0	63.6	0.445
Longitudinal coregistration^a^	—	—	—	—	—	—
Follow‐up, Follow‐up Subspace Projected	47.5	20.0	75.0	28.6	65.2	0.475
Follow‐up, LM‐PCA Projected	60.0	50.0	70.0	45.0	73.7	0.590

a Features based on Ashburner and Ridgway [2013].

Statistically significant balanced accuracy, permutation test *P*‐value < 0.05.

Statistically significant balanced accuracy, permutation test *P*‐value < 0.01.

Here, the classification subject set is disjoint from the longitudinal subject set in the two classification tasks considered: classifying baseline class label with subjects that have only baseline scans and classifying follow‐up class label with subjects that have only follow‐up scans. Note that in this case we cannot form longitudinal features using the longitudinal coregistration method of Ashburner and Ridgway [2013] as each classification set subject has data from only baseline or follow‐up time‐point information.

**Table 3:** OASIS Dataset, Balanced Within‐Set Prediction, discriminating very mild dementia (CDR 0.5) subjects versus HC (CDR 0) subjects with two longitudinal time‐points per subject for all subjects (classification subject set equal to longitudinal subject set in this case)

Features	Bal. accuracy (%)	Sensitivity (%)	Specificity (%)	PPV (%)	NPV (%)	ROC AUC
Predicting Follow‐up Class Label (very mild dementia *n* = 24, HC *n* = 25) using same subjects in longitudinal subject set						
Follow‐up	59.0	50.0	68.0	60.0	58.6	0.627
Longitudinal coregistration^a^	51.2	58.3	44.0	50.0	52.4	0.615
Follow‐up, Baseline Subspace Projected	57.0	50.0	64.0	57.1	57.1	0.552
Follow‐up, Follow‐up Subspace Projected	63.4	70.8	56.0	60.7	66.7	0.700
Follow‐up, LM‐PCA Projected	69.4	70.8	68.0	68.0	70.8	0.718

a Features based on Ashburner and Ridgway [2013].

Statistically significant balanced accuracy, permutation test *P*‐value < 0.05.

Statistically significant balanced accuracy, permutation test *P*‐value < 0.01.

**Table 4:** OASIS Dataset, Unbalanced Within‐Set Prediction, mild and very mild dementia (CDR 1, CDR 0.5) subjects versus HC (CDR 0) subjects with a minimum of three longitudinal time‐points per subject for all subjects (classification subject set equal to longitudinal subject set in this case)

Features	Bal. Accuracy (%)	Sensitivity (%)	Specificity (%)	PPV (%)	NPV (%)	ROC AUC
Predicting Final Follow‐up Class Label (mild and very mild dementia *n* = 14, HC *n* = 28) using same subjects in longitudinal subject set						
Final Follow‐up	53.6	28.6	78.6	40.0	68.6	0.676
Longitudinal coregistration^a^	66.1	42.9	89.3	66.7	75.8	0.653
Final Follow‐up, Baseline Subspace Projected	58.9	35.7	82.1	50.0	71.9	0.686
Final Follow‐up, Final Follow‐up Subspace Projected	50.0	21.4	78.6	33.3	66.7	0.617
Final Follow‐up, LM‐PCA Projected (2 TPs, Short)	53.6	28.6	78.6	40.0	68.8	0.661
Final Follow‐up, LM‐PCA Projected (2 TPs, Long)	62.5	50.0	75.0	50.0	75.0	0.663
Final Follow‐up, LTC‐PCA Projected (All TPs)	67.9	57.1	78.6	57.1	78.6	0.702

a Features based on Ashburner and Ridgway [2013].

Statistically significant balanced accuracy, permutation test *P*‐value < 0.05.

Statistically significant balanced accuracy, permutation test *P*‐value < 0.01.

### Balanced within‐Set Prediction (HNRS Dataset)

As the HNRS has a balanced longitudinal design, with a baseline and follow‐up time‐point made available, we use the balanced version of LM‐PCA, described by Eq. (1) (Balanced Within‐Set Prediction problem using this dataset, predicting the follow‐up time‐point's diagnostic labels). These results are derived from the 24 MCI subjects and 23 HCs with data from both time‐points (follow‐up periods of 2.5 ± 0.2 years). To verify that the improvement we show due to the LM‐PCA projection is not due strictly to dimensionality reduction, we projected cross‐sectional (follow‐up) samples onto cross‐sectional (baseline and follow‐up) subspaces. Table 1 shows the classifier performance measures using unprojected follow‐up features, “longitudinal coregistration” based features [Ashburner and Ridgway, 2013], and follow‐up features projected onto three different subspaces: formed using baseline time‐point information, formed using follow‐up time‐point information and formed using LM‐PCA. In all cases, we performed nested cross‐validation to choose the optimal number of retained PCs in the projection within each outer cross‐validation fold. The LM‐PCA projected features were the only ones whose balanced accuracy exceeded chance (*P* < 0.002, permutation test). In Figure S2 in the Supporting Information, we see across all cross‐validation folds 90% ( k=5 retained PCs) and 95% ( k=16 retained PCs) explained variances were selected to form LM‐PCA projections.

### Balanced Information Transferring Prediction (HNRS Dataset)

Table 2 shows the performance measures obtained when testing the information transfer capability of our approach using the HNRS dataset. Here, we formed a balanced LM‐PCA projection matrix with the subjects used in Table 1 (24 MCI, 23 controls), then predicted the baseline time‐point's diagnostic labels using unprojected and LM‐PCA projected baseline features on a disjoint set of subjects that had a baseline scan but no available follow‐up (79 MCI subjects and 87 controls).^1^ Also shown in Table 2 is the result of predicting the follow‐up time‐point's diagnostic labels using unprojected, follow‐up subspace projected and LM‐PCA projected follow‐up features on a disjoint set of subjects with a follow‐up scan but no corresponding baseline scan (10 MCI subjects and 20 controls) using the same projection matrix (formed with 24 MCI, 23 controls). As each subject has only one time‐point's information in these experiments, we could not form the “longitudinal coregistration” based features in this case as in the other tables. In this case, none of the balanced accuracies statistically exceeded chance under permutation testing.

### Balanced Within‐Set Prediction (OASIS Dataset)

The OASIS dataset has an unbalanced longitudinal design, with each subject scanned on two or more visits. To compare the two time‐point, balanced design LM‐PCA method across datasets we mimicked a balanced longitudinal design with this dataset by restricting the follow‐up times of subjects to be roughly similar to that of the HNRS dataset. We used a subject set composed of subjects with follow‐up scans between 1.4 and 2.5 years after baseline, resulting in follow‐up periods of 1.9 ± 0.3 years (24 subjects with very mild dementia, i.e., CDR 0.5, and 25 HCs, i.e., CDR 0).

Table 3 shows the classifier performance metrics in this case. The LM‐PCA projected features' balanced accuracy exceeded chance (*P* < 0.01, permutation test) while the unprojected follow‐up features' and “longitudinal coregistration” features' balanced accuracies did not. As in Table 1 we also projected follow‐up samples onto subspaces formed using both baseline and follow‐up information. In neither case did the classifier balanced accuracies achieve statistical significance. In Figure S2 in the Supporting Information, we see in this case that the number of PCs explaining mostly 55% and 60% of variance were selected across cross‐validation folds to form LM‐PCA projections, with k=8 and k=9 retained PCs respectively. This contrasts with the higher explained variances (90% and 95%) selected with the HNRS dataset (Table 1), highlighting the need to use nested cross‐validation to tune this parameter.

### Unbalanced Within‐Set Prediction (OASIS Dataset)

In the final experiment, we considered the problem of discriminating those subjects with three or more longitudinal time‐points using the OASIS dataset, without restricting the time interval between samples. To increase the number of disease class subjects, we formed a class consisting of five subjects with mild dementia (corresponding to CDR 1) along with nine subjects with very mild dementia (CDR 0.5), for a total of 14 subjects being discriminated from 28 healthy subjects (CDR 0). There were 33 subjects with three longitudinal measurements, 8 subjects with four longitudinal measurements and one subject with five longitudinal measurements.

We compared the performance of features formed using the cross‐sectional data at the final follow‐up time‐point for each subject (Final Follow‐up) to projecting this data onto five different subspaces: two formed using purely cross‐sectional information (Final Follow‐up, Baseline Subspace Projected and Final Follow‐up, Final Follow‐up Subspace Projected), two formed by performing LM‐PCA using two scans from each subject and one by performing LTC‐PCA using all available scans. The “Final Follow‐up, LM‐PCA Projected (2 TPs, Short)” features were formed by creating an unbalanced LM‐PCA projection [via Eq. (B.3)] using the last two time‐points' information for each subject. In this case the time interval between scans is the shortest possible for each subject, resulting in an interval of 2.0 ± 0.8 years across subjects. The “Final Follow‐up, LM‐PCA Projected (2 TPs, Long)” features were formed in a similar manner by creating an LM‐PCA projection using the first and last time point for each subject, that is, with the longest time intervals between scans for each subject, resulting in an interval of 4.2 ± 1.2 years across subjects. Finally, “Final Follow‐up, LTC‐PCA Projected (All TPs)” were formed using all time‐points for each subject. We used the more general LTC‐PCA for this (with polynomial model order P=1, as it is in LM‐PCA), which allowed us to estimate the slope of samples' longitudinal trajectories using all (three or more) time‐points of each subject rather than having to select two time‐points when using LM‐PCA. As in Table 1 and 3 we also compare to “longitudinal coregistration” features. In this case we coregistered the first and last time‐point of each subject to best compare to the LTC‐PCA projected features.

The performance metrics for all features are shown in Table 4. Classifiers that used the “longitudinal coregistration” and LTC‐PCA projected features were the only ones that statistically exceeded chance under permutation testing. This suggests that when more than two longitudinal samples are available per subject, we can derive a better estimate of the linear coefficient matrix with the more general LTC‐PCA than with the two‐sample LM‐PCA approach.

### Statistical Comparison of Methods

Because of the small sample sizes in each comparison, we have limited statistical power to detect differences between the methods within individual comparisons. Indeed, LM‐PCA/LTC‐PCA projection only improved accuracy relative to unprojected cross‐sectional features or “longitudinal coregistration” based features in one case (LM‐PCA projected vs. unprojected features in Table 1, *P* < 0.05, McNemar's test). However, we are more interested testing whether our method provided a consistent improvement overall (i.e., across all classification problems and datasets) rather than whether it improves individually for any individual classification problem. To assess this, we performed a 3 × 1 repeated measures analysis of variance (ANOVA) with three different methods as the “within‐comparison factor” with the five balanced accuracies across comparisons as the samples for each method. The three methods compared were: (i) the most appropriate version of our method for each contrast (LM‐PCA for the comparisons in Tables 1, 2, 3 or LTC‐PCA for the comparisons in Table 4); (ii) the latest follow‐up samples projected onto the latest follow‐up subspace (the single follow‐up in Tables 1 and 3, the baseline in the first part of Table 2, the follow‐up in the second part of Table 2, the final follow‐up in Table 4) and (iii) features formed using the (unprojected) latest follow‐up samples. The results show a significant difference in the effect of the projection method, with *F*(1.52, 6.08) = 10.91, *P* = 0.01. Post‐hoc pairwise *t*‐tests showed that LM‐PCA/LTC‐PCA projected features performed better overall than unprojected features (*P* = 0.03) and cross‐sectionally projected features (*P* = 0.003) whereas cross‐sectionally projected features did not outperform unprojected features (*P* = 0.74). We could not include “longitudinal coregistration” based features in the ANOVA as it was not possible to form such features in Table 2 due to a lack of either baseline or follow‐up information in the classification subject set. Therefore, we conducted an additional paired *t*‐test, comparing the balanced accuracies of LM‐PCA in Tables 1 and 3 and LTC‐PCA in Table 4 against “longitudinal coregistration” based features' balanced accuracies. This showed no significant differences.

### Discriminative and Forward Brain Maps

**Figure 5:**
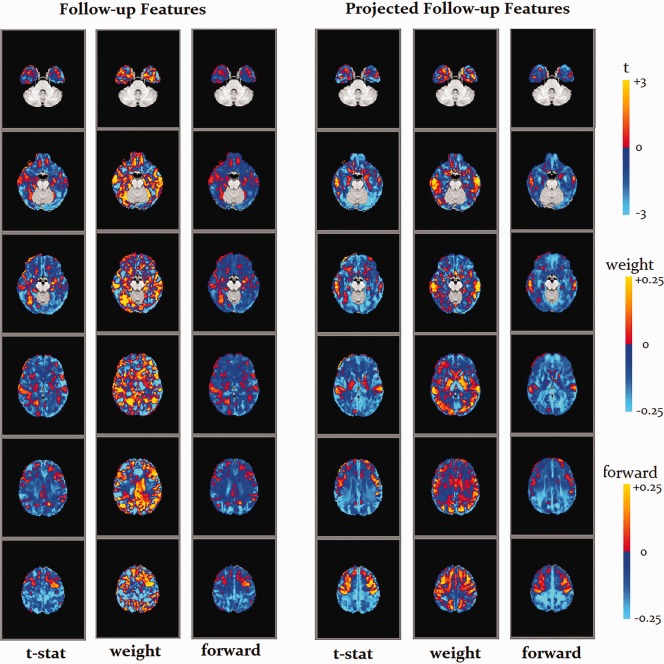
**Comparison of maps for Balanced Within‐Set Prediction problem, HNRS dataset**. Mass univariate differences between groups' features via an (unthresholded) *t*‐statistic and classifier weight and forward maps, discriminating MCI versus HC using cross‐sectional (follow‐up time‐point) features compared to LM‐PCA projected cross‐sectional (same time‐point) features, corresponding to Table 1. Weight and forward maps are scaled such that the maximum absolute value in each image is equal to one. In all maps, positive values (hot colors) depict areas of stronger association to the positive class (MCI group) while negative values (cold colors) depict areas of stronger association to the negative class (HC group).

**Figure 6:**
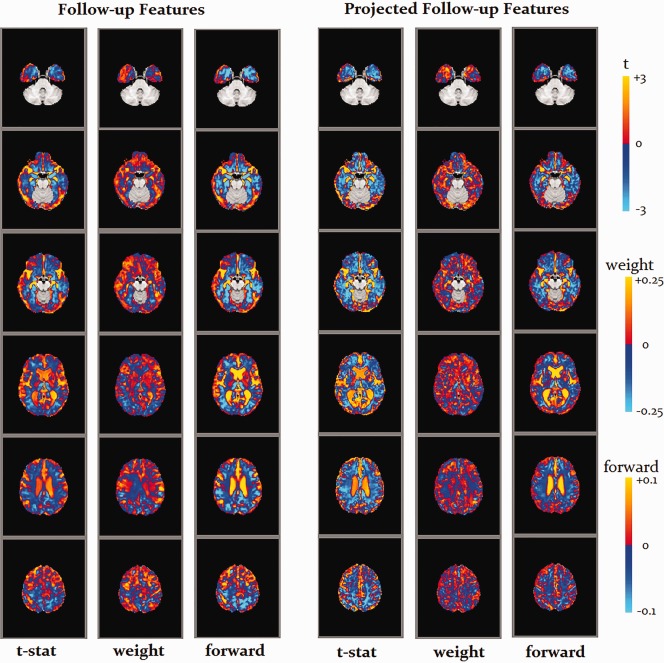
**Comparison of maps for Balanced Within‐Set Prediction problem, OASIS dataset**. Mass univariate differences between groups' features via an (unthresholded) *t*‐statistic and classifier weight and forward maps, discriminating very mild dementia (CDR 0.5) versus HC using cross‐sectional (follow‐up time‐point) features compared to LM‐PCA projected cross‐sectional (same time‐point) features, corresponding to Table 3. Weight and forward maps are scaled such that the maximum absolute value in each image is equal to one. In all maps, positive values (hot colors) depict areas of stronger association to the positive class (CDR 0.5 group) while negative values (cold colors) depict areas of stronger association to the negative class (HC group).

Figures 5 and 6 display *t*‐statistic images, weight maps and forward maps for the cross‐sectional features and LM‐PCA projected cross‐sectional features derived from the Balanced Within‐Set Prediction problem (HNRS and OASIS datasets, respectively). For both types of features, the figures show a good correspondence between the unthresholded two sample *t*‐statistic maps describing group differences between disease subjects and controls at each voxel and forward maps that were generated via Eq. (6). The weight maps depicted, generated via Eq. (5), are useful for understanding which regions are driving the classifier's decisions. However, they differ greatly from the other two map types, reflecting the fact that they do not represent group differences [Haufe et al., 2014].

In Figure 5, which corresponds to the HNRS dataset result in Table 1, we see that the cross‐sectional follow‐up feature‐based forward maps and the LM‐PCA projected counterparts have negative values (cold colors) across many gray and white matter regions. As we are interpreting maps of Jacobian determinant features, quantifying the expansion or contraction of voxels during diffeomorphic registration to an inter‐subject template, areas of negative group differences indicate contraction (i.e., degeneration) in the positive (disease) class relative to the negative (HC) class. In particular, we see the LM‐PCA projected cross‐sectional forward maps have stronger negative values in the parietal and precuneus areas, the frontal pole and the temporal lobe relative to the cross‐sectional map.

In Figure 6, which corresponds to the OASIS dataset result in Table 3, we see that both the cross‐sectional and LM‐PCA projected cross‐sectional forward maps show strong positive values within the lateral ventricles and the Sylvian fissure, indicating a regional shrinkage of brain tissue in early dementia subjects compared to controls. In addition, there are negative values in the temporal lobes in both sets of forward maps. Qualitatively, the two sets of forward maps in this figure resemble each other, with some evidence of de‐noising due to the PCA procedure via a reduction in the number of negative value regions in the LM‐PCA projected cross‐sectional map compared to the purely cross‐sectional forward map. Overall, these patterns of coefficients are highly consistent with the literature on whole brain and ventricular volume change in dementia subjects relative to controls [Freeborough and Fox, 1997; Jack et al., 2004; Nestor et al., 2008].

## DISCUSSION

We have introduced a new feature construction‐based technique for pattern recognition, which makes use of the longitudinal information available in both unbalanced and balanced longitudinal designs. We demonstrated the effectiveness of this technique using high‐dimensional neuroimaging data on the problem of discriminating both MCI subjects from HCs and early dementia subjects from controls, showing improvements in accuracy across two separate datasets. The method introduces a single tuning parameter and, as a feature construction operation, is able to work with existing registration algorithms used in pre‐processing and any pattern recognition algorithm (including regression models). In principle, the method is not limited to neuroimaging and can be applied to any high‐dimensional longitudinal dataset.

We have shown that: (i) in the case of balanced longitudinal designs with two samples per subject, projected cross‐sectional features have higher predictive accuracy in discriminating disease than do unprojected cross‐sectional features; (ii) for unbalanced designs, higher classification accuracy can be attained using three or more samples to estimate the linear coefficient matrix used to form the necessary longitudinal projection compared to using the minimum of two samples and (iii) information transfer from one set of subjects to another is possible using the proposed method. In addition, we have shown that the proposed features, based on a linear transformation, retain the ability to visualize a linear classifier's weight maps and forward maps.

Comparing our method to others in the literature, the improvements we show on the Balanced Within‐Set Prediction problem are similar to those achieved by Chen and DuBois Bowman [2011]. However, a direct comparison is not possible as the authors were discriminating AD from controls using PET data with a 12‐month follow‐up period. The authors report an approximately 10% improvement in predictive accuracy using ROI‐based voxels, similar to what we achieved on the OASIS dataset. Another comparison can be made to Wolz et al. [2010], who created non‐linear manifolds using longitudinal and cross‐sectional information into which they embedded both cross‐sectional and longitudinal data to form features for classification. The authors also considered the problem of discriminating MCI from controls (among other contrasts between AD, MCI, and controls) using T1‐weighted structural MRI. They showed improved discrimination accuracy from 64% using baseline imaging features to 69% using baseline plus longitudinal imaging features.

Ziegler et al. [2015] estimated rates of change (slope) within subjects' samples in a mass‐univariate context, noting that a smaller number of longitudinal samples per subject resulted in reduced parameter accuracy. This is in agreement with the results we present in Table 4, where the “Final Follow‐up, LTC‐PCA Projected (All TPs)” features indicate that improved estimation of the linear coefficient matrix using three or more longitudinal samples per subject leads to better predictions compared to LM‐PCA projected features, which were based on the minimum of two samples per subject (see below).

There appears to be an advantage in using balanced design data rather than unbalanced data when forming longitudinal projections. In Tables 1 and 3 we see that features formed using balanced LM‐PCA projections have statistically significant balanced accuracies while in Table 4 the unbalanced LM‐PCA projected features' balanced accuracies did not achieve significance. This observation is in agreement with Ziegler et al. [2015], who note that less balanced designs led to poorer correspondence of slope estimates with ground truth as well as higher noise levels. When unbalanced design information is available, the result in Table 4 suggests that better predictions result from using all available time‐points' information when forming a longitudinal projection (through the use of LTC‐PCA).

In this article, we have started with the assumption of subject‐specific models of longitudinal trajectories, deriving expressions for the first‐order coefficient (i.e., slope) matrix describing intra‐subject changes over time, across all subjects and all dimensions, for the general case of an unbalanced longitudinal design. The benefit of such an approach is its conceptual and computational simplicity, as one can compute each subject's coefficients once and then assemble the desired coefficient matrix (of any model order) for a particular set of subjects at each cross‐validation fold. Longitudinal trajectories are likely correlated among similarly aged subjects, however, and accounting for these inter‐subject correlations may lead to better estimates of the desired coefficient matrix, potentially improving the Information Transferring Prediction capability of our method.

Accounting for inter‐subject correlations is particularly important for neuroimaging data, where the number of longitudinal samples per subject is often small, amounting to two samples per subject in the HNRS dataset and between two and five in the OASIS dataset. Therefore, for future work we will investigate methods to model such correlations, potentially providing better estimation of subject level coefficients by borrowing strength between subjects. Multi‐task learning models have recently been applied to neuroimaging to account for between‐subject correlations [Marquand et al., 2014; Zhang and Shen, 2012] and may be particularly well suited for this purpose.

We have focussed on building a subspace projection using the matrix of first‐order coefficients. One potential limitation of this decision, imposed by a relatively small number of subjects having full follow‐up data in both datasets, is that the subspace we build is most appropriate for situations where the linear term dominates temporal trajectories. There is evidence that the non‐linear component of trajectories may be important, particularly in older subjects [Fjell et al., 2014; Raz et al., 2010; Raz and Lindenberger, 2011]. To this end, one can estimate separate projections based on the first‐order and second‐order coefficient matrices, selecting the number of PCs retained in each projection using a common or model order specific amount of explained variance, for instance. Features could be then composed of a concatenation of cross‐sectional features projected onto the linear coefficient subspace with features projected onto the quadratic coefficient subspace. Estimating the matrix of second‐order coefficients requires at least three time‐points per subject, with the result in Table 4 suggesting that more than this minimum may be necessary for good estimates. Indeed, the small sample size that results from requiring that all subjects have data in all timepoints is an important limitation of this study. Across the five contrasts we performed, we have shown that there is an improvement due to LM‐PCA/LTC‐PCA projected features relative to strictly cross‐sectional features; however, due to limited number of longitudinal samples, we could not show a consistent improvement within each contrast. Therefore, additional validation of the method on larger samples (such as ADNI^2^) and exploring the conditions under which using higher order coefficient matrices would benefit a predictive model is an important avenue for future work.

In summary, we have introduced a novel means of capitalising on longitudinal information for pattern recognition analysis of high‐dimensional data. We have provided a conceptual framework for modelling longitudinal trajectories of change over time, which enables: (i) the use of balanced and unbalanced longitudinal designs; (ii) the modelling of linear and non‐linear effects; and (iii) the ability to transfer longitudinal information between sets of subjects. Our results suggest that longitudinal subspace projection is a promising method for pattern recognition analysis of longitudinal neuroimaging data.

## Supporting information

Supporting Information

## Footnotes

1 Note that the HNRS is ongoing and many of the follow‐up scans have not yet been made available for analysis.

2 Alzheimer's Disease Neuroimaging Initiative (ADNI) data are available at http://adni.loni.usc.edu.

## APPENDIX A Derivation of Coefficient Matrices for the General Case

Suppose we have a longitudinal training set L with l subjects such that subject i has a variable number of samples $m_{i}$ over time, with total number of samples $M=\overset{l}{\underset{i=1}{\Sigma }}m_{i}$ across all subjects. These samples can be arranged in a temporally ascending sequence ${(x}_{i1},\ldots ,\;x_{im_{i}})$, each sample a D‐dimensional vector in general. We can build models describing the trajectories these samples follow over time, starting from the sample at the initial time, $x_{i1}$, through to the sample at the last available time, $x_{im_{i}}$. We will build these longitudinal trajectory models separately for each sample dimension, assuming a polynomial model of the form

(A.1) $$x(t)=\beta^{\left(0\right)}+\sum_{p=1}^{P}\beta^{\left(p\right)}t^{p}+ɛ$$

where $\beta^{\left(0\right)}$ is a constant term, P is the polynomial function model order, $\beta^{\left(p\right)}$ is the pth order coefficient and ɛ is i.i.d Gaussian measurement error.

The model for all subjects' temporal trajectories can be expressed in the form of a General Linear Model (GLM) as

(A.2) X1X2⋮Xl = Z1Z2⋱Zl B1B2⋮Bl+ E1

where ${\;X}_{i}={\left[x_{i1},\ldots ,x_{{im}_{i}}\right]}^{T}$ is an $m_{i}\times D$ matrix of subject i's samples, ${\;Z}_{i}$ is an $m_{i}\times (P+1)$ longitudinal design matrix which takes the form

(A.3) $${\;Z}_{i}=\left[\begin{array}{cccc} 1 & t_{i1}^{} & \ldots_{}^{} & t_{i1}^{P}\\ 1 & t_{i2}^{} & \ldots_{}^{} & t_{i2}^{P}\\ \vdots_{}^{} & \vdots_{}^{} & \ddots_{}^{} & \vdots_{}^{}\\ 1 & t_{{im}_{i}}^{} & \ldots_{}^{} & t_{{im}_{i}}^{P} \end{array}\right].$$

and $B_{i}$, the (P+1)×D matrix of trajectory coefficients across dimensions, takes the form

(A.4) $${\;B}_{i\;}=\left[\begin{array}{c} b_{i}^{\left(0\right)}\\ \vdots \\ b_{i}^{\left(P\right)} \end{array}\right]$$

where $b_{i}^{\left(p\right)}$ is a row vector containing subject i's pth order coefficients across D dimensions. Finally, $E_{1}$ is an M×D matrix of i.i.d Gaussian measurement errors.

In Eq. (A.2), we make the simplifying assumption that there is no coupling between subjects. As a result, we can solve the GLM independently for each subject, that is:

(A.5) $$B_{i}=Z_{i}^{+}X_{i}$$

where i=1,…, l and $Z_{i}^{+}={\left(Z_{i}^{T}Z_{i}\right)}^{-1}Z_{i}^{T}$ is the pseudo‐inverse of $Z_{i}$. For a chosen polynomial model order P, we can form (P+1) matrices of size l×D, denoted by $B^{\left(0\right)},\ldots ,\;B^{\left(P\right)}$, such that $B^{\left(p\right)}$ contains the coefficients of order p across all subjects. $B^{\left(p\right)}$ is, therefore, formed as

(A.6) $$B^{\left(p\right)}=\left[\begin{array}{c} b_{1}^{\left(p\right)}\\ \vdots \\ b_{l}^{\left(p\right)} \end{array}\right]$$

where the $i^{th}$ row contains the vector $b_{i}^{\left(p\right)}$, corresponding to the (p+1)th row of $B_{i}$, as in Eq. (A.4).

In this article, we use the matrix of first‐order (slope) coefficients across subjects, $B^{\left(1\right)}$, when performing LTC‐PCA. We form $B^{\left(1\right)}$ by first solving (A.5) for each subject, assuming a first‐order model for voxel trajectories ( P=1). We then take the second row of each subject's resulting coefficient matrix (the first‐order coefficients for that subject) and form $B^{\left(1\right)}$ as in Eq. (A.6) when p=1.

## APPENDIX B DERIVATION OF FIRST‐ORDER COEFFICIENTS MATRIX IN THE CASE OF TWO TIME‐POINTS PER SUBJECT

In the case of a longitudinal training set L with two time‐points per subject, we can express the second time‐point as $t_{i2}=t_{i1}+t_{i\Delta }$ for subject i. We can rearrange the model presented in Eq. (A.2) to describe the samples at a given time‐point. At the baseline time‐point the l×D matrix of set L subjects' samples, which we denote by $X_{L}(t_{1})$, can be expressed as

(B.1) $$X_{L}(t_{1})=B^{\left(0\right)}+T_{1}B^{\left(1\right)}+\sum_{p=2}^{P}{T_{1}^{p}B}^{\left(p\right)}+\;E_{2}$$

where $T_{1}$ is an l×l matrix with subjects' first time‐point values $t_{11},\ldots ,t_{l1}$ along the leading diagonal and $E_{2}$ is an l×D matrix of i.i.d Gaussian measurement errors. We can express set L subjects' samples at the second time‐point as

(B.2) $$X_{L}(t_{2})=B^{\left(0\right)}+{(T}_{1}+T_{\Delta })B^{\left(1\right)}+\sum_{p=2}^{P}{{(T}_{1}+T_{\Delta })}^{p}B^{\left(p\right)}+\;E_{2}$$

where $T_{\Delta }$ is an l×l matrix with subjects' time intervals between samples $t_{1\Delta },\ldots ,t_{l\Delta }$ along the leading diagonal. Taking the difference of Eqs. (B.1) and (B.2) and assuming a first‐order model ( P=1) as no higher order model can be estimated in this case,

(B.3) $$D_{L}≜\;T_{\Delta }^{-1}\left(X_{L}\left(t_{2}\right)-X_{L}\left(t_{1}\right)\right)=B^{\left(1\right)}$$

where the diagonal inverse term $T_{\Delta }^{-1}$ is an l×l matrix with $\frac{1}{t_{1\Delta }},\ldots ,\frac{1}{t_{l\Delta }}$ along the leading diagonal. We define the matrix $D_{L}$ as the intra‐subject (longitudinal) differences between the baseline time‐point's samples and the follow‐up time‐point's samples, scaled by the time intervals $t_{i\Delta }$, i=1,…,l. We see that this matrix is proportional to the linear term coefficients of the full time‐course model described in Eq. (A.2).

If we make a stronger assumption of a balanced longitudinal design with two time‐points, such that the follow‐up time‐point takes place a fixed time $t_{\Delta }$ after baseline for all subjects, we have that $T_{\Delta }=t_{\Delta }I$ with I as the l×l identity matrix. We can simplify the difference in Eq. (B.3) to

(B.4) $$D_{L}≜\frac{1}{t_{\Delta }}{(X}_{L}\left(t_{2}\right)-X_{L}\left(t_{1}\right))=B^{\left(1\right)}.$$

In this case, the sample differences, scaled by the time interval between samples, are equal to $B^{\left(1\right)}$. Rather than forming the $B^{\left(1\right)}$ matrix via Eq. (A.6), we can work directly with left hand side of Eq. (B.3) or (B.4), depending on whether the time interval between samples is fixed across subjects (i.e., a balanced design) or is subject specific (i.e., unbalanced design).

## APPENDIX C Method Implementation

We have released an implementation of our method in MATLAB, available at https://github.com/LeonAksman/lpr.

## References

[hbm23317-bib-0001] Anderson VM , Schott JM , Bartlett JW , Leung KK , Miller DH , Fox NC (2012): Gray matter atrophy rate as a marker of disease progression in AD. Neurobiol Aging 33:1194–1202. 21163551 10.1016/j.neurobiolaging.2010.11.001 PMC3657171

[hbm23317-bib-0002] Andersson JLR , Jenkinson M , Smith S (2007a): Non‐linear optimisation. FMRIB Tech Rep TR07JA1. Available at: http://www.fmrib.ox.ac.uk/analysis/techrep/ Last accessed: July 12, 2016.

[hbm23317-bib-0003] Andersson JLR , Jenkinson M , Smith S (2007b): Non‐linear registration aka Spatial normalisation. FMRIB Tech Rep TR07JA2. Available at: http://www.fmrib.ox.ac.uk/analysis/techrep/ Last accessed: July 12, 2016.

[hbm23317-bib-0004] Ashburner J (2007): A fast diffeomorphic image registration algorithm. NeuroImage 38:95–113. 17761438 10.1016/j.neuroimage.2007.07.007

[hbm23317-bib-0005] Ashburner J , Ridgway GR (2013): Symmetric diffeomorphic modeling of longitudinal structural MRI. Brain Imaging Methods 6:197. 10.3389/fnins.2012.00197 PMC3564017 23386806

[hbm23317-bib-0006] Bakkour A , Morris JC , Wolk DA , Dickerson BC (2013): The effects of aging and Alzheimer's disease on cerebral cortical anatomy: Specificity and differential relationships with cognition. NeuroImage 76:332–344. 23507382 10.1016/j.neuroimage.2013.02.059 PMC4098706

[hbm23317-bib-0007] Belhumeur PN , Hespanha JP , Kriegman DJ (1997): Eigenfaces vs. fisherfaces: Recognition using class specific linear projection. IEEE Trans Pattern Anal Mach Intell 19:711–720.

[hbm23317-bib-0008] Bernal‐Rusiel JL , Greve DN , Reuter M , Fischl B , Sabuncu MR , Alzheimer's Disease Neuroimaging Initiative (2013): Statistical analysis of longitudinal neuroimage data with Linear Mixed Effects models. NeuroImage 66:249–260. 23123680 10.1016/j.neuroimage.2012.10.065 PMC3586747

[hbm23317-bib-0009] Bishop CM (2007): Pattern Recognition and Machine Learning. New York: Springer.

[hbm23317-bib-0010] Brodersen KH , Ong CS , Stephan KE , Buhmann JM (2010): The balanced accuracy and its posterior distribution. In: 20th International Conference on Pattern Recognition (ICPR), Istanbul, pp 3121–3124.

[hbm23317-bib-0011] Chang CC , Lin CJ (2011): LIBSVM: A library for support vector machines. ACM Trans Intell Syst Technol 2:27:1–27:27.

[hbm23317-bib-0012] Chen S , DuBois Bowman F (2011): A novel support vector classifier for longitudinal high‐dimensional data and its application to neuroimaging data. Stat Anal Data Min 4:604–611. 25309639 10.1002/sam.10141 PMC4189187

[hbm23317-bib-0013] Chen H‐T , Chang H‐W , Liu T‐L (2005): Local discriminant embedding and its variants, In: IEEE Computer Society Conference on Computer Vision and Pattern Recognition, 2005. CVPR 2005. Vol. 2, pp 846–853.

[hbm23317-bib-0014] Chen R , Resnick SM , Davatzikos C , Herskovits EH (2012): Dynamic Bayesian network modeling for longitudinal brain morphometry. NeuroImage 59:2330–2338. 21963916 10.1016/j.neuroimage.2011.09.023 PMC3254821

[hbm23317-bib-0015] Chu C , Hsu AL , Chou KH , Bandettini P , Lin C (2012): Does feature selection improve classification accuracy? Impact of sample size and feature selection on classification using anatomical magnetic resonance images. NeuroImage 60:59–70. 22166797 10.1016/j.neuroimage.2011.11.066

[hbm23317-bib-0016] Cortes C , Vapnik V (1995): Support‐vector networks. Mach Learn 20:273–297.

[hbm23317-bib-0017] Davatzikos C , Fan Y , Wu X , Shen D , Resnick SM (2008): Detection of prodromal Alzheimer's disease via pattern classification of MRI. Neurobiol Aging 29:514–523. 17174012 10.1016/j.neurobiolaging.2006.11.010 PMC2323584

[hbm23317-bib-0018] Desikan RS , Ségonne F , Fischl B , Quinn BT , Dickerson BC , Blacker D , Buckner RL , Dale AM , Maguire RP , Hyman BT , Albert MS , Killiany RJ (2006): An automated labeling system for subdividing the human cerebral cortex on MRI scans into gyral based regions of interest. NeuroImage 31:968–980. 16530430 10.1016/j.neuroimage.2006.01.021

[hbm23317-bib-0019] Dlugaj M , Weimar C , Gerwig M , Wege N , Verde PE , Dragano N , Moebus S , Jöckel KH , Erbel R , Siegrist J (2010): Prevalence of mild cognitive impairment and its subtypes in the Heinz Nixdorf Recall study cohort. Alzheimers Dement 6:S474. 10.1159/000320988 20956854

[hbm23317-bib-0020] Douaud G , Refsum H , Jager CA , de Jacoby R , Nichols TE , Smith SM , Smith AD (2013): Preventing Alzheimer's disease‐related gray matter atrophy by B‐vitamin treatment. Proc Natl Acad Sci USA 110:9523–9528. 23690582 10.1073/pnas.1301816110 PMC3677457

[hbm23317-bib-0021] Dukart J , Mueller K , Villringer A , Kherif F , Draganski B , Frackowiak R , Schroeter ML (2013): Relationship between imaging biomarkers, age, progression and symptom severity in Alzheimer's disease. NeuroImage Clin 3:84–94. 24179852 10.1016/j.nicl.2013.07.005 PMC3791277

[hbm23317-bib-0022] Fitzmaurice GM , Ravichandran C (2008): A primer in longitudinal data analysis. Circulation 118:2005–2010. 18981315 10.1161/CIRCULATIONAHA.107.714618

[hbm23317-bib-0023] Fjell AM , Westlye LT , Grydeland H , Amlien I , Espeseth T , Reinvang I , Raz N , Dale AM , Walhovd KB (2014): Accelerating cortical thinning: Unique to dementia or universal in aging? Cereb Cortex 24:919–934. 23236213 10.1093/cercor/bhs379 PMC3948495

[hbm23317-bib-0024] Franke K , Gaser C , The Alzheimer's Disease Neuroimaging Initiative (2012): Longitudinal changes in individual BrainAGE in healthy aging, mild cognitive impairment, and Alzheimer's disease. GeroPsych 25:235–245.

[hbm23317-bib-0025] Freeborough PA , Fox NC (1997): The boundary shift integral: An accurate and robust measure of cerebral volume changes from registered repeat MRI. IEEE Trans Med Imaging 16:623–629. 9368118 10.1109/42.640753

[hbm23317-bib-0026] Frisoni GB , Fox NC , Jack CR , Scheltens P , Thompson PM (2010): The clinical use of structural MRI in Alzheimer disease. Nat Rev Neurol 6:67–77. 20139996 10.1038/nrneurol.2009.215 PMC2938772

[hbm23317-bib-0027] Gray KR , Wolz R , Heckemann RA , Aljabar P , Hammers A , Rueckert D (2012): Multi‐region analysis of longitudinal FDG‐PET for the classification of Alzheimer's disease. NeuroImage 60:221–229. 22236449 10.1016/j.neuroimage.2011.12.071 PMC3303084

[hbm23317-bib-0028] Guillaume B , Hua X , Thompson PM , Waldorp L , Nichols TE (2014): Fast and accurate modelling of longitudinal and repeated measures neuroimaging data. NeuroImage 94:287–302. 24650594 10.1016/j.neuroimage.2014.03.029 PMC4073654

[hbm23317-bib-0029] Haufe S , Meinecke F , Görgen K , Dähne S , Haynes JD , Blankertz B , Bießmann F (2014): On the interpretation of weight vectors of linear models in multivariate neuroimaging. NeuroImage 87:96–110. 24239590 10.1016/j.neuroimage.2013.10.067

[hbm23317-bib-0030] He X , Yan S , Hu Y , Niyogi P , Zhang HJ (2005): Face recognition using Laplacianfaces. IEEE Trans Pattern Anal Mach Intell 27:328–340. 10.1109/TPAMI.2005.55 15747789

[hbm23317-bib-0031] Holland D , Dale AM (2011): Nonlinear registration of longitudinal images and measurement of change in regions of interest. Med Image Anal 15:489–497. 21388857 10.1016/j.media.2011.02.005 PMC3115407

[hbm23317-bib-0032] Hua X , Lee S , Yanovsky I , Leow AD , Chou YY , Ho AJ , Gutman B , Toga AW , Jack CR, Jr , Bernstein MA , Reiman EM , Harvey DJ , Kornak J , Schuff N , Alexander GE , Weiner MW , Thompson PM (2009): Optimizing power to track brain degeneration in Alzheimer's disease and mild cognitive impairment with tensor‐based morphometry: An ADNI study of 515 subjects. NeuroImage 48:668–681. 19615450 10.1016/j.neuroimage.2009.07.011 PMC2971697

[hbm23317-bib-0033] Hua X , Lee S , Hibar DP , Yanovsky I , Leow AD , Toga AW , Jack CR, Jr. , Bernstein MA , Reiman EM , Harvey DJ , Kornak J , Schuff N , Alexander GE , Weiner MW , Thompson PM (2010): Mapping Alzheimer's disease progression in 1309 MRI scans: Power estimates for different inter‐scan intervals. NeuroImage 51:63–75. 20139010 10.1016/j.neuroimage.2010.01.104 PMC2846999

[hbm23317-bib-0034] Jack CR , Shiung MM , Gunter JL , O'Brien PC , Weigand SD , Knopman DS , Boeve BF , Ivnik RJ , Smith GE , Cha RH , Tangalos EG , Petersen RC (2004): Comparison of different MRI brain atrophy rate measures with clinical disease progression in AD. Neurology 62:591–600. 14981176 10.1212/01.wnl.0000110315.26026.ef PMC2730165

[hbm23317-bib-0035] Jockel KH (1986): Finite sample properties and asymptotic efficiency of monte carlo tests. Ann Stat 14:336–347.

[hbm23317-bib-0036] Klöppel S , Stonnington CM , Barnes J , Chen F , Chu C , Good CD , Mader I , Mitchell LA , Patel AC , Roberts CC , Fox NC , Jack CR , Ashburner J , Frackowiak RSJ (2008): Accuracy of dementia diagnosis—a direct comparison between radiologists and a computerized method. Brain 131:2969–2974. 18835868 10.1093/brain/awn239 PMC2577804

[hbm23317-bib-0037] Klöppel S , Abdulkadir A , Jack CR, Jr. , Koutsouleris N , Mourão‐Miranda J , Vemuri P (2012): Diagnostic neuroimaging across diseases. NeuroImage 61:457–463. 22094642 10.1016/j.neuroimage.2011.11.002 PMC3420067

[hbm23317-bib-0038] Kraha A , Turner H , Nimon K , Zientek LR , Henson RK (2012): Tools to support interpreting multiple regression in the face of multicollinearity. Front Psychol 3:44. 22457655 10.3389/fpsyg.2012.00044 PMC3303138

[hbm23317-bib-0039] Lemm S , Blankertz B , Dickhaus T , Müller KR (2011): Introduction to machine learning for brain imaging. NeuroImage 56:387–399. 21172442 10.1016/j.neuroimage.2010.11.004

[hbm23317-bib-0040] Leung KK , Ridgway GR , Ourselin S , Fox NC (2012): Consistent multi‐time‐point brain atrophy estimation from the boundary shift integral. NeuroImage 59:3995–4005. 22056457 10.1016/j.neuroimage.2011.10.068

[hbm23317-bib-0041] Marcus DS , Fotenos AF , Csernansky JG , Morris JC , Buckner RL (2010): Open Access Series of Imaging Studies (OASIS): Longitudinal MRI data in nondemented and demented older adults. J Cogn Neurosci 22:2677–2684. 19929323 10.1162/jocn.2009.21407 PMC2895005

[hbm23317-bib-0042] Marquand AF , Brammer M , Williams SCR , Doyle OM (2014): Bayesian multi‐task learning for decoding multi‐subject neuroimaging data. NeuroImage 92:298–311. 24531053 10.1016/j.neuroimage.2014.02.008 PMC4010954

[hbm23317-bib-0043] Misra C , Fan Y , Davatzikos C (2009): Baseline and longitudinal patterns of brain atrophy in MCI patients, and their use in prediction of short‐term conversion to AD: Results from ADNI. NeuroImage 44:1415–1422. 19027862 10.1016/j.neuroimage.2008.10.031 PMC2648825

[hbm23317-bib-0044] Mönninghoff C , Dlugaj M , Kraff O , Geisel MH , Jöckel KH , Erbel R , Weimar C , Wanke I (2015): Are transversal MR images sufficient to distinguish persons with mild cognitive impairment from healthy controls? Acad Radiol 22:1172–1180. 26162248 10.1016/j.acra.2015.04.008

[hbm23317-bib-0045] Morris JC , Storandt M , Miller JP , McKeel DW , Price JL , Rubin EH , Berg L (2001): Mild cognitive impairment represents early‐stage Alzheimer disease. Arch Neurol 58:397–405. 11255443 10.1001/archneur.58.3.397

[hbm23317-bib-0046] Mourão‐Miranda J , Bokde ALW , Born C , Hampel H , Stetter M (2005): Classifying brain states and determining the discriminating activation patterns: Support vector machine on functional MRI data. NeuroImage 28:980–995. 16275139 10.1016/j.neuroimage.2005.06.070

[hbm23317-bib-0047] Nestor SM , Rupsingh R , Borrie M , Smith M , Accomazzi V , Wells JL , Fogarty J , Bartha R , Initiative the ADN (2008): Ventricular enlargement as a possible measure of Alzheimer's disease progression validated using the Alzheimer's disease neuroimaging initiative database. Brain 131:2443–2454. 18669512 10.1093/brain/awn146 PMC2724905

[hbm23317-bib-0048] Nichols TE , Holmes AP (2002): Nonparametric permutation tests for functional neuroimaging: A primer with examples. Hum Brain Mapp 15:1–25. 11747097 10.1002/hbm.1058 PMC6871862

[hbm23317-bib-0049] Orrù G , Pettersson‐Yeo W , Marquand AF , Sartori G , Mechelli A (2012): Using Support Vector Machine to identify imaging biomarkers of neurological and psychiatric disease: A critical review. Neurosci Biobehav Rev 36:1140–1152. 22305994 10.1016/j.neubiorev.2012.01.004

[hbm23317-bib-0063] Raz N , Ghisletta P , Rodrigue KM , Kennedy KM , Lindenberger U , (2010): Trajectories of brain aging in middle‐aged and older adults: Regional and individual differences. NeuroImage 51: 501–511. 20298790 10.1016/j.neuroimage.2010.03.020 PMC2879584

[hbm23317-bib-0050] Raz N , Lindenberger U (2011): Only time will tell: Cross‐sectional studies offer no solution to the age‐brain‐cognition triangle—Comment on. Psychol Bull 137:790–795. 21859179 10.1037/a0024503 PMC3160731

[hbm23317-bib-0051] Risacher SL , Saykin AJ , West JD , Shen L , Firpi HA , McDonald BC (2009): Baseline MRI predictors of conversion from MCI to probable AD in the ADNI cohort. Curr Alzheimer Res 6:347–361. 19689234 10.2174/156720509788929273 PMC2764863

[hbm23317-bib-0052] Schmermund A , Möhlenkamp S , Stang A , Grönemeyer D , Seibel R , Hirche H , Mann K , Siffert W , Lauterbach K , Siegrist J , Jöckel KH , Erbel R (2002): Assessment of clinically silent atherosclerotic disease and established and novel risk factors for predicting myocardial infarction and cardiac death in healthy middle‐aged subjects: Rationale and design of the Heinz Nixdorf RECALL Study. Am Heart J 144:212–218. 12177636 10.1067/mhj.2002.123579

[hbm23317-bib-0053] Smith SM , Jenkinson M , Woolrich MW , Beckmann CF , Behrens TEJ , Johansen‐Berg H , Bannister PR , De Luca M , Drobnjak I , Flitney DE , Niazy RK , Saunders J , Vickers J , Zhang Y , De Stefano N , Brady JM , Matthews PM (2004): Advances in functional and structural MR image analysis and implementation as FSL. NeuroImage 23:S208–S219. 15501092 10.1016/j.neuroimage.2004.07.051

[hbm23317-bib-0054] Stang A , Moebus S , Dragano N , Beck EM , Möhlenkamp S , Schmermund A , Siegrist J , Erbel R , Jöckel KH , Heinz Nixdorf Recall Study Investigation Group (2005): Baseline recruitment and analyses of nonresponse of the Heinz Nixdorf Recall Study: Identifiability of phone numbers as the major determinant of response. Eur J Epidemiol 20:489–496. 16121757 10.1007/s10654-005-5529-z

[hbm23317-bib-0055] Studholme C , Cardenas V , Blumenfeld R , Schuff N , Rosen HJ , Miller B , Weiner M (2004): Deformation tensor morphometry of semantic dementia with quantitative validation. NeuroImage 21:1387–1398. 15050564 10.1016/j.neuroimage.2003.12.009

[hbm23317-bib-0056] Turk M , Pentland A (1991): Eigenfaces for recognition. J Cogn Neurosci 3:71–86. 23964806 10.1162/jocn.1991.3.1.71

[hbm23317-bib-0057] Wolfers T , Buitelaar JK , Beckmann CF , Franke B , Marquand AF (2015): From estimating activation locality to predicting disorder: A review of pattern recognition for neuroimaging‐based psychiatric diagnostics. Neurosci Biobehav Rev 57:328–349. 26254595 10.1016/j.neubiorev.2015.08.001

[hbm23317-bib-0058] Wolz R , Aljabar P , Hajnal JV , Rueckert D (2010): Manifold learning for biomarker discovery in MR imaging In: Wang F, Yan P, Suzuki K, Shen D, editors. Machine Learning in Medical Imaging, Lecture Notes in Computer Science. Springer Berlin Heidelberg, pp 116–123.

[hbm23317-bib-0059] Zhang D , Shen D (2012): Multi‐modal multi‐task learning for joint prediction of multiple regression and classification variables in Alzheimer's disease. NeuroImage 59:895–907. 21992749 10.1016/j.neuroimage.2011.09.069 PMC3230721

[hbm23317-bib-0060] Zhao D , Lin Z , Xiao R , Tang X (2007): Linear Laplacian discrimination for feature extraction. In: IEEE Conference on Computer Vision and Pattern Recognition, Minneapolis, MN, pp 1–7.

[hbm23317-bib-0061] Ziegler G , Penny WD , Ridgway GR , Ourselin S , Friston KJ (2015): Estimating anatomical trajectories with Bayesian mixed‐effects modeling. NeuroImage 121:51–68. 26190405 10.1016/j.neuroimage.2015.06.094 PMC4607727

[hbm23317-bib-0062] Zipunnikov V , Greven S , Shou H , Caffo B , Reich DS , Crainiceanu C (2014): Longitudinal high‐dimensional principal components analysis with application to diffusion tensor imaging of multiple sclerosis. Ann Appl Stat 8:2175–2202. 25663955 10.1214/14-aoas748 PMC4316386

