# Supplementary material for: Making use of longitudinal information in pattern recognition
Source: Hum Brain Mapp. 2016 Jul 25;37(12):4385–404. doi: 10.1002/hbm.23317 (PMC5111621; doi:10.1002/hbm.23317)
Supplement: Supplementary file 1 — Supporting Information [file HBM-37-4385-s001.docx]

**Making use of longitudinal information in pattern recognition**

***Supplementary Information***

**Supplementary Materials and Methods**

*MCI Diagnostic Criteria for the Heinz-Nixdorf Recall substudy (HNRS)*

A standardized neuropsychological examination was conducted by a neuropsychologist using the following test assessments: Alzheimer’s Disease Assessment Scale (ADAS), the number connection test of the Nuremberg Gerontopsychological Inventory (German: Nürnberger Altersinventar (NAI)) (Oswald and Fleischmann, 1994), the verbal fluency test (Aschenbrenner et al., 2000) and an Instrumental Activities of Daily Living (IADL) (Oswald and Fleischmann, 1994) scale to assess activities of daily living. For each cognitive domain age-specific test norms were administered. A cognitive domain was rated as impaired if the performance was more than 1 standard deviation (SD) below the age adjusted mean. A cut-off of 1 SD was chosen for the screening test as well as for the detailed neuropsychological assessment, because it was found to be associated with a higher relative prognostic power in predicting the development of dementia compared with a cut-off of 1.5 SD (Busse et al., 2003). Furthermore it provides a higher sensitivity, which was particularly important for the screening test to detect participants in need of further neuropsychological assessment (Busse et al., 2003). Depression was assessed using the depression subscale of the ADAS (Ihl and Weyer, 1993). A detailed physical examination with particular focus on the neurological examination was conducted by a neurologist. Furthermore, a medical history was gathered related to cognitive functioning, duration of such symptoms, history of other medical illnesses and current treatment.

MCI was diagnosed according to the International Working Group on MCI criteria (Petersen, 2004; Winblad et al., 2004) with the exception of the cognitive complaint criterion (the subject or the informant had to express some concern about the person’s cognitive function). Thus, the following criteria were necessary for the diagnosis: (1) evidence for impairment in cognitive function on the administered objective cognitive tasks, which was not normal for age; (2) evidence for preserved basis activities of daily living/minimal impairment in complex instrumental functions and (3) exclusion of the DSM-IV (Diagnostic and Statistical Manual of Mental Disorders, fourth edition) dementia diagnosis. Participants with dementia, severe depression (ADAS depression subscale score >4), Parkinson’s disease, mental retardation, severe alcohol consumption (for women > 20g/day; for men >40g/day), known brain cancer, severe problems with the German language (in foreign persons) and severe sensory impairment leading to invalid cognitive testing were excluded from the analysis.

**Supplementary Results**

*Effect of SVM C Parameter Variation*

In the classification we present in Tables I-IV we fixed the value of support vector classifier’s $C$ parameter at $C=1$ throughout. This choice was made based our experience using SVC with very high-dimensional feature vectors derived from MRI. In Figure S1 we tested this choice by assessing classifier performance at values of $C$ between ${10}^{-5}$ and ${10}^{5}$ with logarithmic spacing. We considered the same classification problem presented in Table I: the *Balanced Within-Set Prediction* problem of discriminating MCI subjects from healthy controls using the HNRS dataset, with two time-points per subject.

Figure S1 depicts the classifier balanced accuracies at these values of $C$ for the three types of features we compared: follow-up time-point information, longitudinal (Ashburner and Ridgway, 2013), and proposed LM-PCA projected follow-up time-point information. For all three types of features we observe stability in classifier performance between $C=0.1$ and $C={10}^{5}$, with the cross-sectional follow-up and longitudinal features’ performance remaining unchanged and the projected follow-up features’ varying between 70% and 75%. The chosen value of $C=1$, depicted by the dashed line, falls within this stable region. We observed similar stability of classifier performance at $C=1$ in the other classifications we performed.


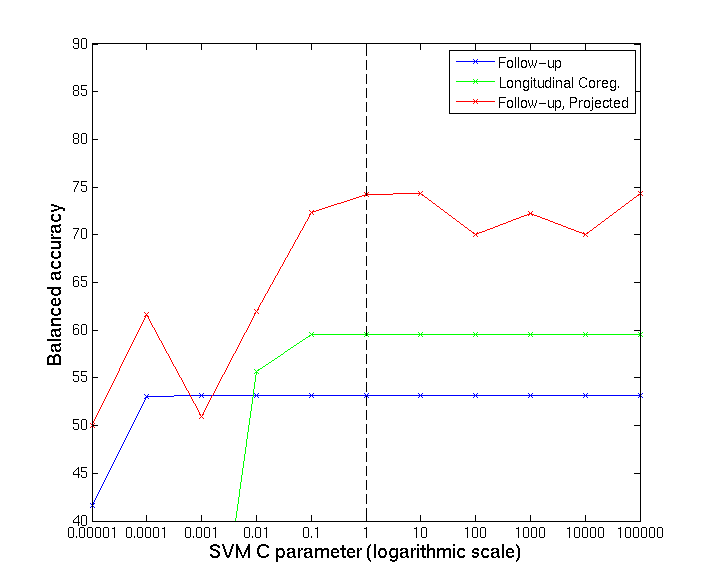


**Figure S1.** Effect of varying SVM $C$ parameter on balanced accuracy of classifying MCI subjects versus Healthy Controls (HC) in HNRS dataset (*Balanced Within-Set Prediction* problem considered in Table I). The $C=1$ point shown as dashed line corresponds to the fixed value used in all results presented.

*Optimized Explained Variance Percentage and Corresponding Number of Principal Components*

In all relevant experiments, we performed nested cross-validation to choose $k$, optimal number of retained principal components (PCs) within each cross-validation fold used to form the matrix $\boldsymbol{U}_{k}$ necessary for creating the longitudinal subspace projection $\boldsymbol{U}_{k}{\boldsymbol{U}_{k}}^{T}$. At each nested fold we varied the amount of variance explained as described in the *Nested Cross-Validation* section of the Materials and Methods. In Figure S2 we show the distribution of chosen explained variance percentage across cross-validation folds for the two *Balanced Within-Set Prediction* problems we considered in Tables I and III. For both datasets, we also show the corresponding number of retained PCs associated with a given amount of explained variance. As the number of retained PCs needed for a given amount of explained variance may differ at each nested cross-validation fold, for each outer fold we calculated the median number of retained PCs across all nested (inner) folds, which turned out, in both classifications, to be a unique number across all outer folds for a given amount of explained variance.

We see that the two distributions differ greatly: the classifier exclusively chooses 90% and 95% explained variances when discriminating MCI subjects from controls using the HNRS dataset (Table I), with $k=5$ and $k=16$ corresponding retained PCs respectively. When discriminating very mild dementia subjects from controls using the OASIS dataset (Table III), the explained variances are mostly 55% and 60% with $k=8$ and $k=9$ retained PCs respectively. The distributions overlap at 90% explained variance, with $k=31$ associated PCs in the OASIS case and $k=5$ associated PCs in the HNRS case.

**
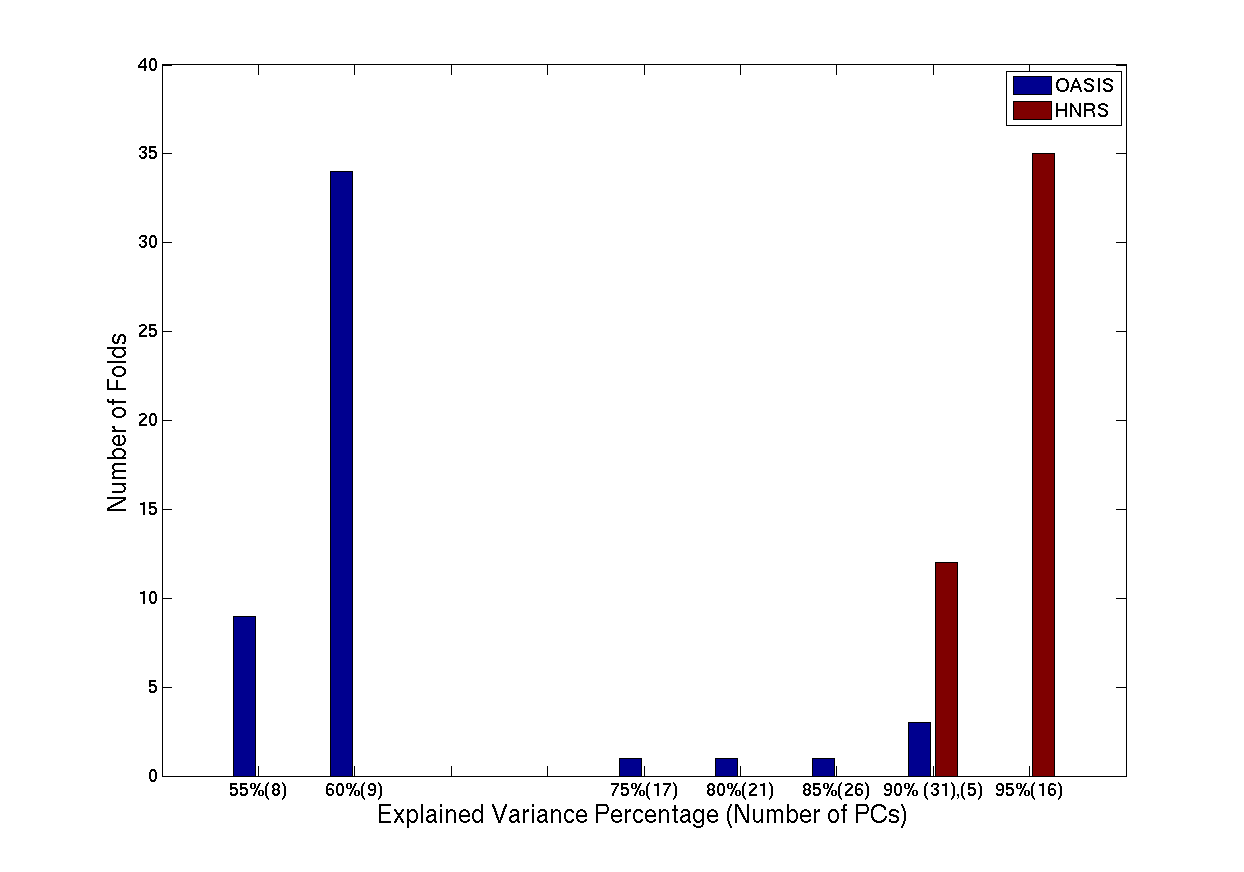
Figure S2.** Histogram of number of cross-validation folds with given explained variance percentage and corresponding number of principal components for the *Balanced Within-Set Prediction* classification problem, discriminating MCI subjects vs. HCs using HNRS dataset (Table I result, shown in red bars) and discriminating very mild dementia from HCs using the OASIS dataset (Table III result, shown in blue bars).

**References**

Aschenbrenner S., Lange K.W., Tucha O., 2000. RWT: Regensburger Word Fluency Test. Hogrefe Verlag für Psychologie, Göttingen.

Ashburner, J., Ridgway, G.R., 2013. Symmetric diffeomorphic modeling of longitudinal structural MRI. Brain Imaging Methods 6, 197. doi:10.3389/fnins.2012.00197

Busse, A., Bischkopf, J., Riedel-Heller, S.G., Angermeyer, M.C., 2003. Subclassifications for mild cognitive impairment: prevalence and predictive validity. Psychol. Med. 33, 1029–1038.

Ihl R., Weyer, G., 1993. Alzheimer’s disease assessment scale. Hogrefe Verlag für Psychologie, Göttingen

Oswald W., Fleischmann U.M., 1994. Nuremberg Age Inventory (NAI), Hogrefe Verlag für Psychologie, Göttingen.

Petersen, R.C., 2004. Mild cognitive impairment as a diagnostic entity. J. Intern. Med. 256, 183–194. doi:10.1111/j.1365-2796.2004.01388.x

Winblad, B., Palmer, K., Kivipelto, M., Jelic, V., Fratiglioni, L., Wahlund, L.-O., Nordberg, A., Bäckman, L., Albert, M., Almkvist, O., Arai, H., Basun, H., Blennow, K., De Leon, M., DeCarli, C., Erkinjuntti, T., Giacobini, E., Graff, C., Hardy, J., Jack, C., Jorm, A., Ritchie, K., Van Duijn, C., Visser, P., Petersen, R. c., 2004. Mild cognitive impairment – beyond controversies, towards a consensus: report of the International Working Group on Mild Cognitive Impairment. J. Intern. Med. 256, 240–246. doi:10.1111/j.1365-2796.2004.01380.x
